# Streamlining the isolation of fungal hyphae: a semi-automated approach for soil substrates

**DOI:** 10.1093/jxb/eraf539

**Published:** 2025-12-16

**Authors:** Isabelle Elisabeth Metzen, Marcel Bucher

**Affiliations:** Institute for Plant Sciences, Cologne Biocenter, University of Cologne, Zuelpicher Straße 47b, D-50674 Cologne, Germany; Institute for Plant Sciences, Cologne Biocenter, University of Cologne, Zuelpicher Straße 47b, D-50674 Cologne, Germany; Lancaster University, UK

**Keywords:** Hyphal length density, hyphoplane, hyphoplane microbiome, hyphosphere, microbe–microbe interactions, optimization of fungal hyphae isolation, sieving and sucrose centrifugation, soil microbial communities

## Abstract

Extracting fungal hyphae with their naturally associated microbiota from soil samples presents a significant challenge due to their small size, typically in the micrometer range, and the formation of dynamic fungal networks. We combined elements of previous protocols and automated the wet-sieving steps of the methodology to efficiently extract fungal hyphae from various soil types, including natural loamy soils. This approach reduces manual handling, minimizes operator-dependent variability, and shortens processing time by up to 2.5-fold. Unlike earlier methods that require sand or glass bead supplementation, which can introduce artificial conditions and limit large-scale field applications, our sieving and sucrose centrifugation (SSC) method avoids these drawbacks. The SSC technique both enables quantification of hyphal length density (HLD) and, importantly, preserves surface-associated microbes for downstream analyses. Among the tested methods, SSC yielded the highest HLD. Using a combination of microscopy, molecular techniques, and next-generation sequencing (NGS), we demonstrate that this method allows targeted study of bacteria tightly attached to fungal hyphae. Furthermore, the SSC approach effectively enriched fungal hyphae from a highly diverse soil community, establishing a dependable tool for advancing research on fungal hyphae as microbial hotspots in soil ecosystems.

## Teaser

We introduce the sieving and sucrose centrifugation (SSC) technique, which combines, optimizes, and semi-automates existing methods to efficiently extract diverse fungal hyphae from natural soils and preserve attached microbes.

## Introduction

Plant tissues and their immediate surroundings host a diverse array of microorganisms, including bacteria, protists, viruses, oomycetes, and fungi. Most fungi in natural soils form hyphae, filamentous structures that extend and branch throughout the soil. These hyphae play a crucial role in nutrient acquisition, facilitating the uptake and transport of essential elements. They also contribute to the spread of fungal networks, support spore formation, and enable the colonization of plant roots, thereby shaping soil microbial communities and plant–fungal interactions. Some fungi, such as yeasts, either completely lack hyphae or have them in a highly reduced form, living primarily as single cells (Singara Charya, 2015). These non-hyphal fungi make up only a small fraction of the total fungal biomass in soil, whereas hyphal-forming fungi dominate and play a crucial role in decomposing organic matter and driving nutrient cycling (Treseder and Lennon, 2015; van der Heijden *et al*., 2015). Additionally, some fungi, such as *Mucor* species, exhibit dimorphism, switching between yeast-like and hyphal forms in response to environmental conditions. This ability enhances their adaptability, allowing them to colonize diverse habitats and interact more effectively with hosts (Boyce and Andrianopoulos, 2015).

Among filamentous fungi which colonize plant roots, arbuscular mycorrhizal fungi (AMF) of the subphylum Glomeromycotina are well studied, while fungi of the subphylum Mucoromycotina have been more recently explored (Orchard *et al*., 2017). Fungi from both taxa form mutualistic symbioses with their plant host and extend their extraradical hyphae into the soil, accessing nutrient patches beyond the root system of the plant. They absorb nutrients such as phosphorus (P) in the form of inorganic orthophosphate (Pi) or nitrogen (N) and deliver them to plant roots in exchange for carbon in the form of sugars and lipids (Pfeffer *et al*., 1999; Trépanier *et al*., 2005; Spatafora *et al*., 2016; Jiang *et al*., 2017; Keymer *et al*., 2017; Prout *et al*., 2024). The full diversity of fungi and associated microbes capable of directly providing nutrients for plant uptake remains incompletely understood.

Dependence on interactions with soil bacteria for Pi uptake, due to limited ability to mineralize soil organic P, has been demonstrated particularly for AMF (Tisserant *et al*., 2013; Zhang *et al*., 2014; Wang *et al*., 2017). AMF extraradical hyphae (ERH) extend into the soil, guiding bacteria to nutrient-rich patches, and enhance nutrient acquisition (Zhang *et al*., 2018; Jiang *et al*., 2021).

Although only a subset of soil bacteria attach to or enter fungal hyphae (Toljander *et al*., 2007; Scheublin *et al*., 2010), microbial analyses often lack consistent distinctions between the hyphal compartments: the hyphosphere (soil influenced by ERH), the hyphal endosphere (inside hyphae), and the hyphoplane (hyphal surface). Bacterial communities at the hyphoplane differ significantly from those in the hyphosphere and bulk soil (Gahan and Schmalenberger, 2015; Zhang *et al*., 2018; Wang *et al*., 2019, 2022; Zhou *et al*., 2020; Emmett *et al*., 2021). However, it remains unclear how far the hyphal sphere of influence extends into the surrounding soil and differentiates microbial communities. Early work in 1991 suggested that fungal hyphae can alter pH and P levels in their vicinity (Li *et al*., 1991). This raised the question of whether hyphal effects extend far enough to differentiate the microbial community in the hyphosphere, not just in the hyphoplane, from the bulk soil. Insights into this question emerged from a few studies, highlighting that the hyphosphere varies in fungal and bacterial community composition compared with bulk soil (Nuccio *et al*., 2013; Xu *et al*., 2018; Emmett *et al*., 2021; Qin *et al*., 2022). Thus, hyphal presence has a detectable impact on microbial composition in the surrounding soil volume, appropriately termed the hyphosphere. A study investigating all three compartments—hyphoplane, hyphosphere, and bulk soil—further confirmed that the bacterial community in the hyphosphere differs in the abundance of *Burkholderiaceae*, *Sandaracinaceae*, *Chloroflexi*, and *Acidibacter* from that in the bulk soil (Emmett *et al*., 2021).

Cross-kingdom research, particularly focusing on the interactions between fungal hyphae and bacteria, is an emerging field of research that has in the past received relatively little attention, probably due to the inherent difficulty of isolating hyphae, that typically range in size from 2 µm to 20 µm, from natural substrates. So far, only a limited number of studies have explored whether roots and hyphae assemble distinct bacterial and fungal communities, despite strong indications that such differences exist. For instance, Zhang *et al*. (2024) reported that roots and AMF hyphae host distinct bacterial communities. However, the underlying mechanisms determining the extent to which plant genotypic variation influences these microbial communities remain largely unknown. One possible explanation could be differences in hyphal secretory activities, which may shape microbial assemblages in unique ways.

In field, greenhouse, and *in vitro* experiments aimed at characterizing microbial communities assembled by fungal hyphae, a compartmented system with a small-pore, root-impermeable nylon mesh is commonly used to separate roots from hyphae (Jakobsen and Rosendahl, 1990). These hyphal compartments are essential for studying extraradical hyphae and their associated microbial communities without the confounding influence of roots.

Historically, methods for extracting fungal hyphae from soil began in the late 1940s, when Jones *et al*. (1948) mixed soil samples into molten agar, spread them thinly on slides, and stained them for microscopic analysis. Later, various approaches such as manual hyphal picking under the stereomicroscope, a highly labor-intensive and time-consuming method, as well as sieving, blending, or centrifugation were developed or modified.

Hanssen *et al*. (1974) introduced a filter-based method, in which blended soil was filtered, washed, and stained for quantifying hyphae of *Trichoderma viride*. Abbott *et al*. (1984) refined this technique to specifically extract AMF, reducing the processing time and making the method suitable for fluorescent staining. Jakobsen *et al*. (1992) further adapted this approach for sandy soils using blending and filtration, introducing minor improvements.

However, these blending and sieving methodologies are not suitable for loamy or clay-rich soils, as filters clog due to the high content of fine soil particles. To overcome this, many studies have incorporated sand or glass beads into the soil of root-free compartments to simplify hyphal extraction (e.g. Jakobsen *et al*., 1992; Wang *et al*., 2019; Zhou *et al*., 2020; Emmett *et al*., 2021; Zhang *et al*., 2024). Nevertheless, adding such components to the soil is impractical for large-scale field applications and creates artificial conditions that disrupt the natural plant–microbe–soil continuum. For example, the addition of sand to loamy soils alters texture, bulk density, pH, water retention, and nutrient availability, which can affect plant nutrient uptake, root gene expression, and carbon allocation (Vetterlein *et al*., 2020; Ganther *et al*., 2022). In addition, the contrast in surface roughness between smooth glass beads and natural soil particles can influence microbial colonization behavior (Long and Or, 2007).

In contrast, Bingle and Paul (1986) developed a method combining wet-sieving with sucrose density gradient centrifugation that allowed hyphae and spore extraction even from clay and loamy soils. This method resembles the sucrose-based spore isolation technique by Allen *et al*. (1979), designed for extracting spores from soil. However, the so-called differential water/sucrose centrifugation method (WSCM) has disadvantages: numerous filtration steps and a centrifugation time of 40 min per sample batch limit its throughput. Furthermore, the use of sodium pyrophosphate in this method negatively affects DNA amplification, as it inhibits Taq polymerase activity (Johnson *et al*., 1995), which is problematic for studies aiming to analyze microbial communities associated with fungal hyphae.

More recently, Awad and Pena (2023) modified the WSCM method to extract hyphae from silty clay, silty clay loam, and loamy sand by omitting sodium pyrophosphate and significantly reducing the procedure time. However, the authors acknowledged that to achieve higher extraction efficiency, their method requires an additional pre-treatment step, involving the manual removal of fine root fragments and other debris under the stereomicroscope. This step is time-consuming, operator-dependent, and limits the suitability of the method for high-throughput or large-scale studies.

For none of the existing diverse methodologies is it known whether they preserve microbial attachment to fungal hyphae, which is essential for studying microbial interactions at the hyphoplane across different soil types. Additionally, critical steps such as manual wet-sieving are highly operator-dependent, introducing variability in extraction efficiency and reducing reproducibility, especially in large-scale studies.

After hyphal extraction, hyphal length density (HLD) can be manually measured using the visual gridline intersection (VGI) method (Newman, 1966; Tennant, 1975; Shen *et al*., 2016) or semi-automated tools such as the Fiji plug-in AnaMorf (Barry *et al*., 2009; Schindelin *et al*., 2012) or the HyLength software (Cardini *et al*., 2020), respectively.

Here, we introduce a semi-automated sieving and sucrose centrifugation (SSC) method for efficiently extracting fungal hyphae with attached microbes directly from various natural soils, including clay-rich field sites. Building on the protocols of Bingle and Paul (1986) and Jakobsen *et al*. (1992), the SSC approach combines automated high-amplitude wet-sieving to effectively remove loamy soil with manual sucrose density gradient centrifugation to concentrate hyphae and spores. By automating the critical sieving step, the method minimizes operator-dependent variability, improves reproducibility, and increases throughput compared with conventional manual approaches. Furthermore, this method avoids the use of sand or glass bead supplementation, reducing artificial disturbance, and preserves the natural microbial communities attached to fungal hyphae. This enables reliable hyphal length quantification as well as the identification of microbes closely associated with fungal hyphae, providing a robust tool for advancing hyphoplane microbiome research. In addition to developing the SSC workflow, we evaluated the performance of previously described hyphal counting methods relative to the SSC approach, outlining their respective advantages and limitations. Ultimately, we employed the SSC method to characterize the fungal and bacterial hyphoplane microbiome and demonstrate how its taxonomic composition differs from that of the surrounding hyphosphere and bulk soil.

## Materials and methods

### Set-up for Experiment 1: method development

To develop the SSC method for the efficient extraction of fungal mycelium, we used a substrate consisting of a quartz/soil mixture previously planted with *Allium schoenoprasum* and inoculated with *Rhizophagus irregularis* spores (DAOM 197198). This substrate contained a high density of fungal mycelium, making it well-suited for method optimization.

### Set-up for Experiment 2: hyphal extraction and hyphal length assessment of field samples via the SSC method

**Fig. 1.**
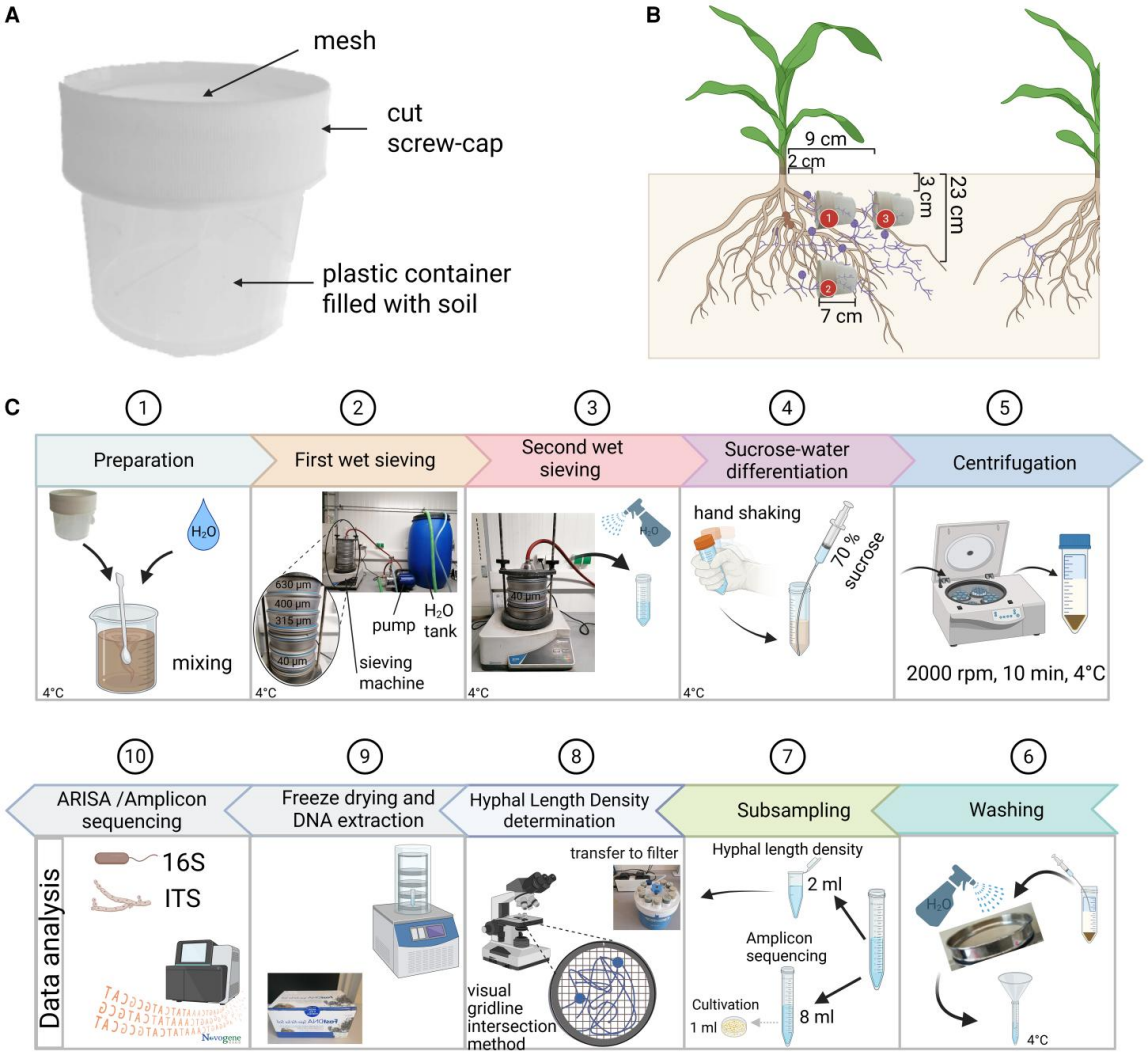
Diagrams showing the design of the compartmented system, fungal hyphae enrichment procedure, and hyphae-associated microbiome analysis. (A) Schematic representation of the hyphal containers (HCs), consisting of plastic containers with modified screw lids. The central part of each lid was cut out, a mesh was placed over the opening of the container and tightly sealed by screwing on the cut lid. (B) Schematic representation illustrating the positioning of HCs containing natural soil, fungal spores and mycelium, and microorganisms, but excluding roots, in the field. Each 7 cm long plastic container creates individual hyphae-associated compartments. The containers are placed horizontally at two distances from the center of the root system and at two different depths in the soil for each plant. The figure is not to scale. (C) Stepwise procedure for the isolation of hyphae from natural field soil. HCs were harvested and processed semi-automatically using the SSC method at 4 °C. The method includes steps 1–10. Created in BioRender. Bucher, M. (2025) https://BioRender.com/njshm9v.

For Experiment 2, soil containing fungal hyphae was harvested from three field experiments conducted in 2019, 2020, and 2021 at the University of Cologne Field Station, located at coordinates 50.925617°N, 6.936641°E. Field plots were allocated for NK (–P), PK (–N), or NPK (full) fertilized silty loam. The field plots differ in their nutrient status and were thus used to examine whether patterns of hyphal colonization correlate with root colonization under varying nutrient conditions. To spatially separate hyphae-associated compartments (hyphosphere, hyphoplane, and hyphal endosphere) from root-associated compartments (rhizosphere, rhizoplane, and root endosphere), hyphal containers (HCs) were used. This set-up is based on a previously described compartmented system used to quantify phosphate uptake in mycorrhizal plants (Nagy *et al*., 2005). The cylindrical plastic containers (Thermo Fisher Scientific, USA; 10077900 X12 jar, 125 ml PP, straight-walled, autoclavable) were filled with field soil from the respective field area and modified by excising the center of the screw cap. A 21 μm nylon mesh was then placed over the container opening and securely kept in place covering the opening by screwing the cut lid back on, with the mesh oriented toward the plant root (Fig. 1A). This set-up allowed fungal hyphae to grow into the container while preventing root intrusion. To prevent root growth around or beneath the lid and ensure a tight seal, the cap was additionally sealed with parafilm. This set-up ensures that microbial communities associated with fungal hyphae can be studied independently of direct root influence. Three HCs per plant were inserted at two depths (topsoil, 3–10 cm; and subsoil, 23–30 cm) and two distances (2 cm and 9 cm) from the root system center (Fig. 1B). The HCs were collected during the vegetative stage of *Zea mays*. To obtain the hyphosphere sample, the natural soil inside the HC was collected. The hyphoplane sample was isolated using the SSC method, while bulk soil was sampled from an unplanted area of the field.

### Extraction of fungal hyphae (SSC method)

For both experiments, the SSC method was used to extract fungal hyphae from direct soil substrates or from soil within HCs (Fig. 1C). The method includes two automated wet-sieving steps with manual sample loading, sucrose centrifugation, washing steps, and sample collection. The entire sieving process and sample handling were performed in a cold room set to 4 °C. In detail, 4 °C-cold deionized water was pumped through a low-pressure water pump into the wet-sieving machine AS 200 Basic (Retsch, Haan, Germany), which included a water-blast pipe and a stacked sieving convoy consisting of a 630 µm sieve (ISO 3310/1, 200×50 mm, made of stainless steel), a 400 µm sieve, a 315 µm sieve, an aeration ring, a 40 µm sieve, and a drainage ring. The water pressure was set to 2.5 bar, and the amplitude was adjusted to 75% of the maximum available power setting, which corresponds to an ∼2.25 mm oscillation amplitude at a fixed frequency of 50 Hz, as per the specifications of the AS 200 Basic model. Soil samples weighing between 25 g and 300 g were placed in a beaker and the beakers were filled to 400 ml with deionized water at 4 °C. For individual experiments, we recommend using soil samples of similar weight to ensure consistency. The samples were then mixed manually with a spoon to facilitate the detachment of fungal hyphae from dense soil particles. The tested and validated sample sizes for the SSC method are represented by these ranges: the protocol was not applied to samples smaller than 25 g or larger than 300 g.

After mixing, the samples were manually placed on the first sieve of the convoy for processing. The samples underwent an initial automated sieving process lasting 8 min. The first three sieves with the widest mesh sizes of 630, 400, and 315 µm were manually removed, and the remaining material was sieved automatically for an additional 6 min. Following sieving, hyphae retained on the 40 μm sieve were manually washed into 50 ml microcentrifuge tubes, resulting in slight variations in sample volume. For standardization, each tube was adjusted to a final volume of 20 ml with deionized water, ensuring a 1:1 ratio of aqueous sample to sucrose solution. The solutions were shaken by hand to resuspend the particles; alternatively, the samples could be vortexed. Equal volumes of 70% sucrose were then carefully injected beneath the aqueous solution using a syringe fitted with a 7 cm long plastic tube (0.5 cm diameter) and the samples were centrifuged at 805 *g* for 10 min at 4 °C. Hyphae and spores were collected from the interface between the two solutions using the same syringe and plastic tubing. The plastic tubing was carefully inserted into the interface, and the hyphae were gently aspirated together with the surrounding H_2_O and sucrose solution. The tubing was held at the interface while suction continued until both solutions were completely collected, taking care not to destroy any bottom pellets. This minimizes the risk of hyphae loss and reduces operator-dependent variability. The collected material was then transferred onto a 40 μm sieve and gently washed multiple times with deionized water using a spray bottle to prevent the loss of hyphae remaining on the sieve, then collected in a clean centrifuge tube. The final volume was adjusted to 10 ml to standardize the volume across all samples for downstream analysis.

### Manual estimation of hyphal length density (VGI method)

To measure HLD (fungal hyphae in centimeters per hyphal container) in both experiments, 2 ml of the solution containing hyphae and spores was filtered onto a nitrocellulose membrane (pore size: 1.2 μm) using a manifold holding system connected to a vacuum pump. A vacuum was applied to remove excess liquid from the filter. The retained hyphae and spores were then stained by immersing the filter in an ink solution [5% acetic acid, 5% ink (Pelikan 4001 royal blue)] for 20 min. After staining, the ink solution was aspirated through the vacuum, and the sample was rinsed with ddH_2_O. Once dried, the intersections of hyphae with the gridlines were counted at 13 sites per sample under a Leica DM 1000 LED microscope at ×10 magnification. This gridline intersection counting is based on the manual method described by Newman (1966) and Tennant (1975). The HLD was subsequently calculated following the methods of Tennant (1975) and Shen *et al*. (2016). Determining the HLD in the bulk soil, specifically the soil used to fill the HCs but located outside of them, provides a means to quantify background HLD that is independent of the experimental treatment.

### Comparison of methods for hyphal length counting

In Experiment 1, we compared two semi-automated methods for HLD quantification, the AnaMorf plugin for Fiji (Barry *et al*., 2009, 2015; Schindelin *et al*., 2012) and the HyLength software (Cardini *et al*., 2020), with the manually performed VGI method (Newman, 1966; Tennant, 1975; Shen *et al*., 2016). Two approaches were chosen.

#### Approach A

Fungal hyphae and spores were extracted using the SSC method from different sample sizes (100, 50, and 25 g) of a substrate rooted by *A. schoenoprasum*, which provided sufficient AMF hyphae for analysis. This substrate is referred to as QSF. HLD counts were obtained using the manual VGI method and the two automated methods (AnaMorf and HyLength) across 18 matched samples per method, covering complete, half, and quarter sample amounts. For AnaMorf analysis, grayscale images (8-bit) were generated in Fiji, and image resolution was set to 1, maximum branch length to 10, maximum circularity to 1, and minimum area to 10. Results were exported as CSV files. HyLength was used following the developers’ recommendations (Cardini *et al*., 2020). HLD was compared using a paired *t*-test. In addition, the mean absolute error (MAE) and mean percentage error (MPE) were calculated by comparing the HLD of each method with the manually determined counts, using the manual values as the reference standard.

#### Approach B

To determine the quantitative object-level accuracy by assessing false positives and false negatives of both automated methods (AnaMorf and HyLength) relative to manual annotation, seven representative hyphal extraction images were selected. These were captured using a Leica DM1000 LED microscope and Leica Application Suite Version 2.8.1. The accuracy of the automated methods was assessed by comparing the detected *x*, *y* coordinates with manually annotated hyphal midpoints in Fiji, which served as the ground truth. For AnaMorf, a log file containing detection coordinates was generated. An R script was used to extract only valid *x* and *x* coordinates of detected objects and filter out all detections labeled ‘object does not meet morphological criteria’, leaving only valid object positions for analysis. For HyLength, detection coordinates were approximated by calculating the midpoint of each skeleton branch using Fiji’s ‘Analyze Skeleton’ plugin, as, to our knowledge, the software does not provide direct *x*, *y* coordinates through its user interface. A detection was considered a true positive if it fell within a 10 pixel radius of a manual annotation. Automated detections without a nearby manual match were counted as false positives, and manual points with no corresponding detection were considered false negatives. Based on these classifications, we calculated precision (TP/[TP+FP]), recall (TP/[TP+FN]), and the F1 score as the harmonic mean of precision and recall.

### Comparison with the aqueous blending and sieving method and the differential water/sucrose centrifugation method

In Experiment 1, the hyphal yield obtained via the SSC method was compared with that obtained using the aqueous blending and sieving method (BSM) as described by Jakobsen *et al*. (1992), and the WSCM (Bingle and Paul, 1986). Hyphae and spores were extracted from 80 g of a quartz/soil mixture following the respective protocols, and HLD was measured using the VGI method.

In the BSM, we followed the protocol of Jakobsen *et al*. (1992), with the only deviation being the use of ink stain (5% acetic acid, 5% ink) instead of trypan blue. In brief, the quartz/soil mixture was blended with 250 ml of ddH_2_O for 30 s, briefly shaken, and allowed to settle for 1 min. A 2 ml supernatant sample was then taken for HLD measurement.

In accordance with the protocol of Bingle and Paul (1986), with minor adjustments to the equipment available in our laboratory, soil samples were dispersed in the WSCM using a mixer at maximum speed for 2 min in 500 ml of 0.1% sodium pyrophosphate. The resulting suspension was passed through stacked sieves with mesh sizes of 140, 200, and 315 µm. The material retained on the sieves was then repeatedly washed with 0.1% sodium pyrophosphate to remove soil particles, and subsequently concentrated to 5 ml. This concentrated hyphal suspension was then applied to a continuous glycerol density gradient (0–100% v/v glycerol over a 15 ml glycerol cushion in a 100 ml centrifuge tube) and centrifuged for 40 min at 113 *g* and 4 °C to separate the hyphae from the residual soil.

For the SSC method [see ‘Extraction of fungal hyphae (SSC method)’], soil from HCs was, in brief, mixed with 400 ml of ddH_2_O, passed through sequential sieves, and subjected to sucrose centrifugation as described above. Hyphae and spores were collected from the interface, thoroughly washed, and transferred to a 15 ml tube for further processing and analysis.

### DNA extraction and microbiome sequencing

In Experiment 2, an 8 ml aliquot of the aqueous suspension containing hyphae, obtained through the use of the SSC method, was freeze-dried. DNA was extracted from the dried hyphae and spores using the Fast DNA Spin Kit for Soil (MP Biomedicals, Eschwege, Germany) according to the manufacturer’s protocol, with the modification that homogenization using the Precellys 24 tissue lyzer (Betin Technologies, Montigny-le-Bretonneux, France) was performed twice, each for 30 s at 6200 rpm. ITS2 amplicons for fungi were generated using the ITS3 (GCATCGATGAAGAACGCAGC) and ITS4 (TCCTCCGCTTATTGATATGC) primer pair, while 16S V4 amplicons were obtained using the 515F (GTGCCAGCMGCCGCGGTAA) and 806R (GGACTACHVGGGTWTCTAAT) primer pair (White *et al*., 1990; Caporaso *et al*., 2011). PCR amplification, sample quality control, and library generation were performed by the sequencing provider Novogene (Munich, Germany). In general, six replicates per condition and controls (H_2_O, empty kit control) were sequenced on the NovaSeq PE250 instrument, with a sequencing depth of 50 000 raw reads per sample. The sequencing reads were demultiplexed, and primers and barcodes removed by the sequencing provider.

The raw sequences were processed with the DADA2 pipeline v1.8.0 and the phyloseq package in RStudio, to obtain amplicon sequencing variants (ASVs) (McMurdie and Holmes, 2013; Callahan *et al*., 2016). The forward and reverse bacterial reads were truncated at 180 bp and 170 bp, respectively [truncLen=c(180,170)], with a maximum of one error in forward and reverse reads [maxEE=c(1,1)] and a default quality score of two (truncQ=2). For fungal reads, no fixed length truncation was applied. Filtered reads were de-replicated and de-noised. Next, forward and reverse reads were merged into ‘contig’ sequences, and chimeras were removed. The fungal taxonomy was assigned using the UNITE database (version UNITE general FASTA release for eukaryotes 9.0) (Abarenkov et al., 2024) and for bacterial taxonomy assignment using the SILVA Database (version 138.1) (Quast et al., 2013). ASVs corresponding to mitochondria, chloroplasts, archaea, and eukaryotes were removed from the bacterial ASVs, and ASVs associated with mitochondria and chloroplasts were removed from the fungal ASVs using the subset_taxa function from the phyloseq package. Although a large number of bacterial ASVs were detected, even with strong filter settings, rarefying was not performed as rare species would have been excluded from the analysis. In the negative controls, zero sequencing output was reported by the provider, or the sequencing percentage of identified ASVs was significantly lower, and the number of chimeric sequences was significantly higher compared with other environmental samples. For beta diversity analysis (Bray–Curtis dissimilarity index), the phyloseq:distance function from the phyloseq package was employed. Bray–Curtis dissimilarities were visualized through principal coordinates analysis (PCoA) using the dudi.pco and s.class functions available in the ade4 package (Dray and Dufour, 2007). Permutational ANOVA (PERMANOVA) on the Bray–Curtis distance matrix was conducted with the adonis2 function from the vegan package (*P*<0.05 and permutations=9999) (Oksanen *et al*., 2007). To assess the relative abundance of individual taxa, the number of sequences attributed to each specific taxon was divided by the total number of sequences and multiplied by 100.

### Scanning electron microscopy

To verify bacterial attachment to hyphae after applying the SSC method in Experiment 2, samples were analyzed using a scanning electron microscope (FEI Quanta 250 FEG) at 20 kV. To further confirm bacterial presence on AMF hyphae, two additional approaches were employed: (i) PCR-based detection using the 515F and 806R primer pair (see above) and subsequent gel electrophoresis; and (ii) DAPI staining (Coleman, 1980).

### General statistics and figure generation

All statistical analyses were conducted in RStudio (version 1.4.1). Data distribution was assessed using the Shapiro–Wilk test of normality (Shapiro.test function) (Royston, 1982, 1995). Depending on the distribution, statistical tests were selected, including ANOVA followed by Tukey’s HSD test, the Wilcoxon test, or the Kruskal–Wallis test (Bauer, 1972; Hollander and Wolfe, 1973; de Mendiburu, 2019). The agricolae package in R was used for ANOVA with Tukey’s test (*P*<0.05) and the Wilcoxon test [*P*<0.05, false discovery rate (FDR) corrected]. Figures were generated using the ggplot function from the ggplot2 package (Wickham, 2016) for data visualization. Correlograms were produced using the ggcor function in ggplot2, based on Spearman correlation. Venn diagrams were generated by using the ggvenn_pq function from the MiscMetabar package (R package version 0.14.1) (Taudière, 2023). Figures were further refined and finalized in Inkscape (v.0.294) for improved presentation. Schematic illustrations were designed using BioRender to ensure clarity and consistency.

## Results and discussion

We developed the SSC method by combining, optimizing, and semi-automating previously described methodologies with two primary objectives: (i) to efficiently extract fungal hyphae from natural soils; and (ii) to preserve the structural integrity of both hyphae and spores for the subsequent study of their associated microbial communities.

### Experiment 1: development of the SSC method

#### Optimization of SSC method parameters

In the initial step, optimal parameters for the automated sieving steps, such as water pressure and sieving amplitude, were determined using a quartz/soil mixture, which contained a high abundance of fungal hyphae (see the Materials and methods). An amplitude >75% of the maximum available power (corresponding to ∼2.25 mm oscillation amplitude at 50 Hz), combined with a water pressure of 3.0 bar, resulted in up to 100% spore damage and 18% hyphal damage (Supplementary Fig. S1A). A water pressure of 2.5 bar was sufficient to extract hyphae while minimizing water consumption. An amplitude of 75% led to the most effective sieving outcome. Based on these findings, the parameters were set to 2.5 bar water pressure and 75% amplitude. The amount of remaining soil organic matter and residual material (including sand, roots, and other debris) varied depending on soil type, with sandy soils leaving more residual material than soils with higher clay content (Supplementary Fig. S2). This is consistent with a previous report showing that sandy soils often yield greater amounts of coarse residual material, such as roots and sand particles, during wet-sieving, in contrast to clay-rich soils (Cambardella and Elliott, 1992).

#### Effect of gravel on hyphal integrity

Since field soil often contains stones with diameters of up to 15 mm, classified as medium gravel (USDA Soil Taxonomy size classification (Soil Survey Staff, 1975), and was not pre-filtered before being added to the hyphal containers in our experiment, we tested the mechanical impact of medium gravel on the integrity of hyphae and spores. To do so, medium gravel was incorporated into a stone-free QSF substrate containing sand (Supplementary Fig. S1B). Despite the presence of gravel, both hyphae and the majority of fungal spores remained intact, indicating that the removal of gravel prior to initiating the sieving process is not required. In agreement, fungal spores are thick walled and have previously been shown to resist mechanical stress, maintaining structural integrity during standard wet-sieving and even under ultrasound-assisted wet-sieving protocols (Boyno *et al*., 2023).

#### Minimizing bacterial contamination

The effectiveness of UV light treatment, hypochlorite treatment, and sonication (35 kHz, 5 min), followed by rinsing with water, in preventing bacterial contamination from residual material of previously sieved samples was evaluated using sieves intended for subsequent sample processing. PCR-based detection targeting the V4 region of the 16S rRNA gene was used to assess bacterial and archaeal contamination. Amplification results showed no detectable 16S rRNA signal in DNA extracted from water that had passed through rinsed sieves, indicating that sonication followed by water rinsing was the most effective method for minimizing bacterial contamination (Supplementary Fig. S3).

#### Comparison of the SSC method with previously described methods

**Fig. 2.**
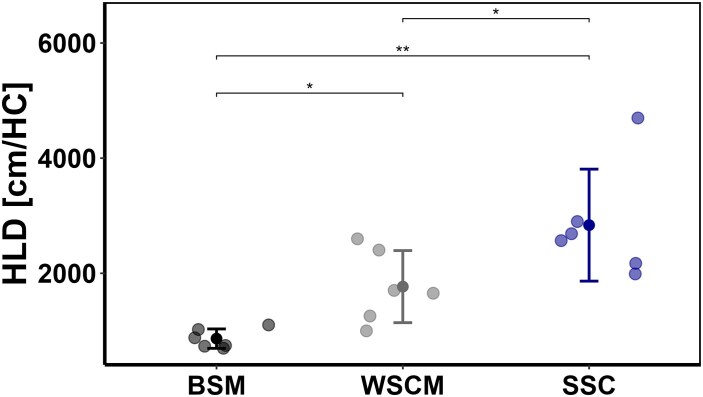
Comparison of fungal hyphal length density (HLD) in hyphal containers (HCs) obtained by three different isolation methods. Soil from three HCs was mixed and again separated into three equal parts. Fungal hyphae within these subsamples were extracted using either the BSM (Jakobsen *et al*., 1992), the WSCM (Bingle and Paul, 1986), or our newly developed sieving and sucrose centrifugation (SSC) method. HLD was then measured using the VGI method. Statistical analysis was conducted using one-way ANOVA followed by Tukey’s HSD test (*P*<0.05, *n*=6). Significance codes: 0.001 ‘**’ 0.01 ‘*’.

A comparison of the SSC method with the previously established BSM and WSCM (Bingle and Paul, 1986; Jakobsen *et al*., 1992) demonstrated that the SSC method yields a higher amount of extraradical hyphae from field soil (Fig. 2). The SSC protocol combines automated sieving with manual centrifugation and handling, enabling the processing of 10 samples in ∼2.2 h, compared with 4.7 h with the WSCM and 2.5 h with the BSM. This represents a substantial reduction in processing time relative to traditional protocols. Furthermore, automation of the sieving step within SSC reduces operator-dependent variability, enhances reproducibility across samples, and lowers contamination risk due to fewer manual handling steps. The SSC method is also less technically demanding, making it suitable for routine, higher throughput applications. Taken together, while classical methods remain suitable for small-scale separations, the SSC approach offers a faster, scalable, and cold-controlled alternative optimized for DNA-sensitive studies and microbial community analyses, with high throughput.

To evaluate the accuracy of semi-automated hyphal length measurement methods, we compared the Fiji plug-in AnaMorf and the HyLength software against manual annotation using the VGI method, which is generally considered the most accurate approach. We calculated the MAE and MPE for the Fiji plug-in AnaMorf and the HyLength software across all sample amounts (complete, half, and quarter).

For complete samples, AnaMorf showed very large deviations from manual counts, with an MAE of 3350.84 cm and an MPE of 228.44%. An MPE of 228.44% means the estimate is, on average, ∼3.28 times larger than the reference value, indicating substantial overestimation. On the other hand, HyLength performed better, with an MAE of 1830.74 cm and an MPE of 118.02% (Supplementary Fig. S4). In half samples, AnaMorf again produced the largest errors (MAE 3957.61 cm, MPE 456.75%), while HyLength showed substantially lower deviations (MAE 719.84 cm, MPE 84.04%). In quarter samples, AnaMorf continued to strongly overestimate hyphal length (MAE 3405.49 cm, MPE 1072.19%), whereas HyLength remained more accurate, with an MAE of 552.72 cm and an MPE of 162.25%. Overall, HyLength consistently produced smaller errors than AnaMorf, indicating that it more reliably approximates manual counts across all sample sizes.

Cardini *et al*. (2020) previously compared manual measurements with AnaMorf and HyLength for measuring *R. irregularis* hyphae *in vivo*. They found that these estimates depend heavily on the fungal species and hyphal density, so the results are only partially comparable with our study, in which we measured the hyphal length of a diverse fungal community under natural soil conditions. In their work, HyLength slightly overestimated hyphal length by ∼21 cm per meter, corresponding to an MPE of 21%, which is consistent with our results. In line with our data, they also showed that HyLength is more accurate than AnaMorf. Interestingly, their study reported that AnaMorf underestimates hyphal length, whereas we observed a significant overestimation, which is likely to be due to differences in sample type and user-specific handling.

Both automated methods tended to overestimate hyphal length compared with manual annotation, particularly for smaller sample amounts. In addition to its higher accuracy, the VGI method also demonstrated the most effective scalability: hyphal counts across full, half, and quarter samples followed the expected decreasing order, a pattern not consistently observed in the automated methods (Supplementary Fig. S4). These results support VGI as the most robust method under our experimental conditions.

To complement the count-based evaluation, an object-level analysis was conducted to estimate the number of false positives and false negatives relative to the manual annotations. For AnaMorf, the mean values across seven images were: precision=0.013, recall=0.118, and F1 score=0.025. On average, for every correctly identified true-positive finding, ∼77 false positives were detected, and eight true positives were overlooked (false negatives). These results show that, although some structures were correctly identified in our hands, the method also produced numerous false positives and overlooked a significant proportion of true hyphae.

For the HyLength software, object-level detection could not be reliably assessed because, to our knowledge, the *x*, *y* coordinates of detected objects cannot be exported from the user interface. The workaround using the skeleton image (see the Materials and methods) introduced a spatial bias that persisted despite parameter adjustments. Consequently, the resulting values would not accurately reflect the true precision of the HyLength software due to these technical constraints.

In sum, however, it can be said that all results show that although both semi-automatic methods have limitations in accurately detecting hyphal structures, the manual VGI method remains the most reliable and scalable approach to quantifying hyphal length under the present experimental conditions.

### Experiment 2: hyphal extraction and assessment of field samples via the SSC method

#### Hyphal distribution and hyphal length density correlations

**Fig. 3.**
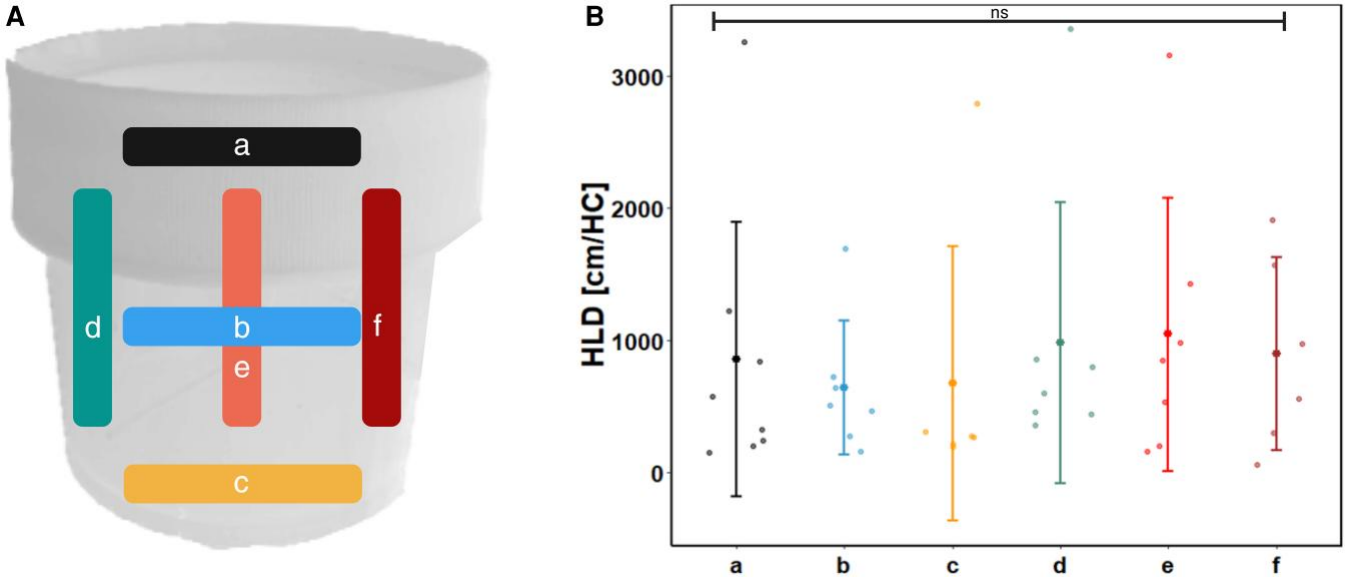
Hyphal length density (HLD) is evenly distributed within the hyphal container (HC). The HCs harvested from the field in 2019 were divided into either three horizontal subsamples [top (A), middle (B), bottom (C)] or three vertical subsamples [left (D), middle (E), right (F)]. HLD within these subsamples was determined by the SCC and VGI methods. Different letters indicate significant differences between HLD in different positions (one-way ANOVA followed by Tukey’s HSD test, *P*<0.05, *n*=5–7). Significance codes: 0.05 ‘ns’, not significant. Created in BioRender. Bucher, M. (2025) https://BioRender.com/3r1ukeu.

To assess the distribution of hyphae within the hyphal container, samples were collected from six distinct positions, spanning both horizontal and vertical orientations (Fig. 3). Analysis using the SSC method revealed an even distribution of hyphae, which is essential when working with subsamples. While previous studies in asymbiotic systems, such as that of Richter *et al*. (2024), focusing on single-species *R. irregularis* cultures in microfluidic devices, have reported localized hyphal responses including growth arrest, septation, and edge-tracking upon encountering physical obstacles, our findings do not indicate similar accumulation at container boundaries. Importantly, our system differs in two key aspects: it is a symbiotic system, where fungal growth is influenced by dynamic interactions with host roots; and it contains diverse microbial communities, including but not limited to, AMF. The growth dynamics and spatial behavior of these diverse microbial taxa under symbiotic conditions remain poorly understood, and their responses to spatial constraints may differ substantially from those observed in simplified, asymbiotic model systems. This highlights the need for further research into species- and community-level growth patterns in ecologically realistic settings.

**Fig. 4.**
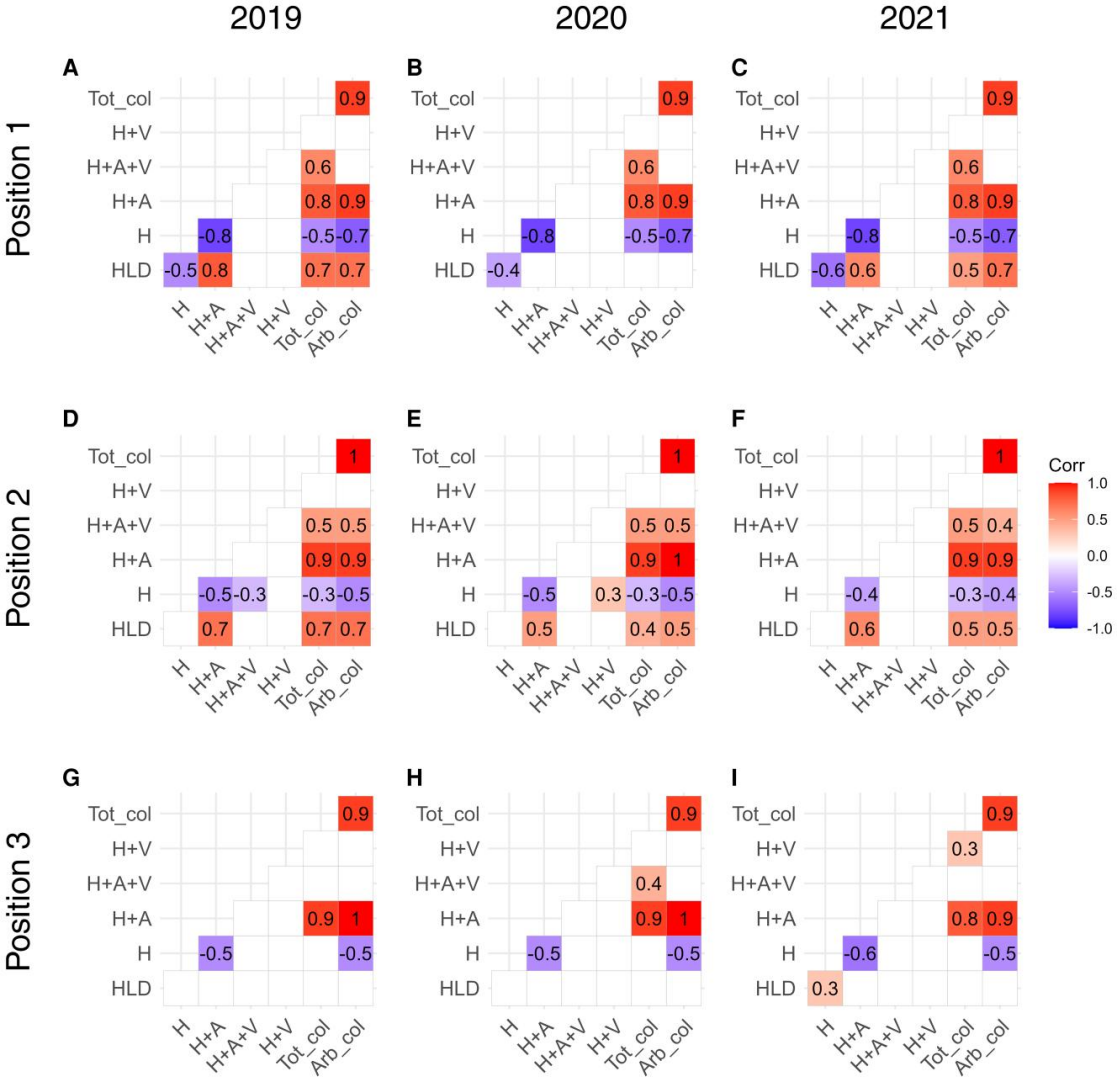
Hyphal length density (HLD) correlates with the presence of hyphae and arbuscules (H+A). Hyphal containers were harvested from wild-type *Zea mays* plants in their vegetative stage grown on NK (-P), PK (-N), or NPK fields in 2019, 2020, and 2021, and HLD within the hyphal containers was determined using the SCC and VGI methods. Fungal structures in roots were classified under the microscope into one of the following categories: hyphae alone (H), hyphae with vesicles (H+V), hyphae with arbuscules (H+A), arbuscules, vesicles, and hyphae (H+A+V), or non-colonized (no-myc). Total colonization (Tot_col) was calculated as the sum of all categories excluding no-myc, whereas arbuscule colonization (Arb_col) was calculated by including only categories with arbuscules. The percentage of fungal structures was correlated with HLD and with each other. Correlations are presented separately for each field year and for the containers at positions 1–3. Only significant correlations (*P*<0.05) are shown, with numbers indicating Spearman’s rank correlation coefficient. Correlations range from negative to positive, as indicated in the scale on the right. *n*=18. Created in BioRender. Bucher, M. (2025) https://BioRender.com/3f64bf1.

**Fig. 5.**
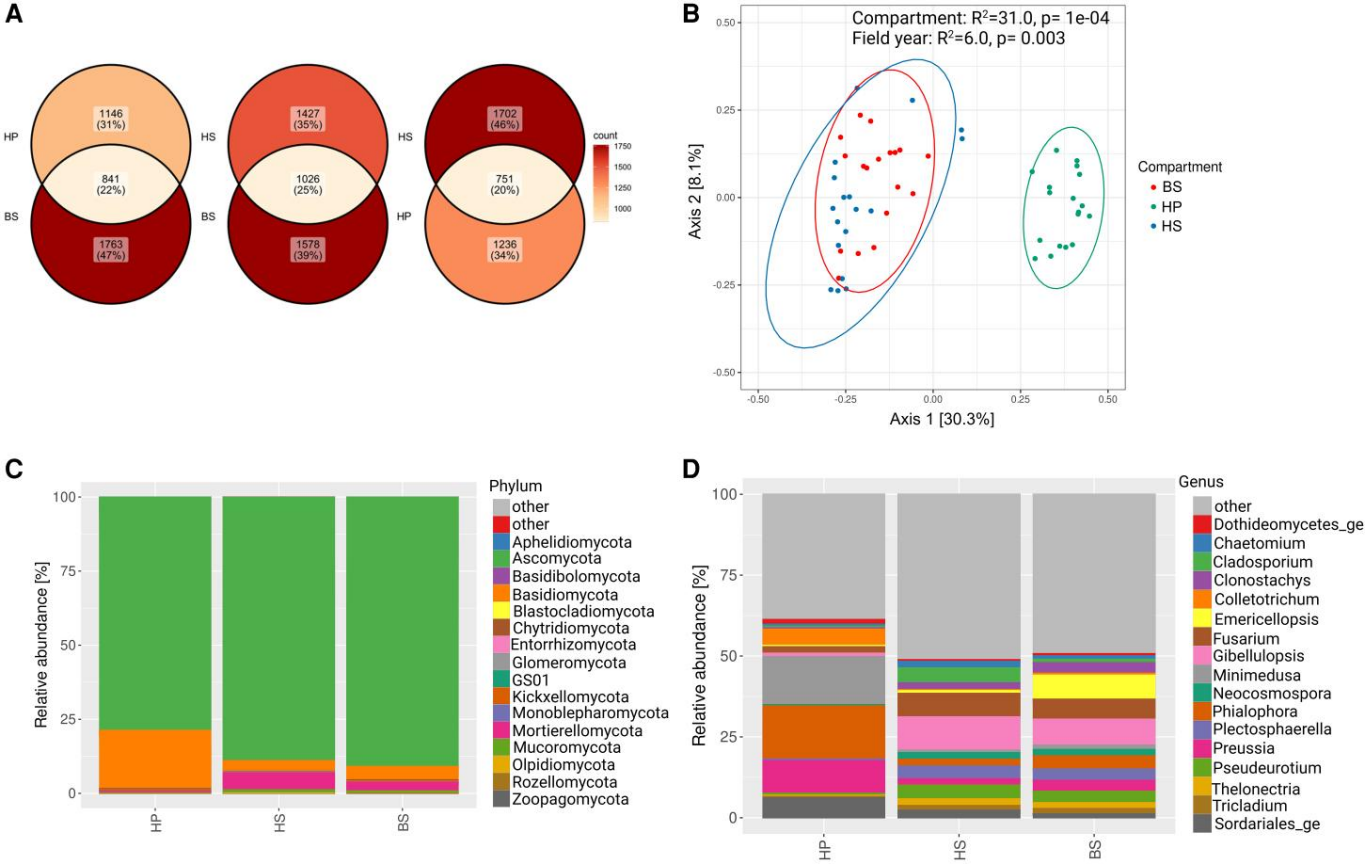
The SSC approach enriched fungal hyphae from a highly diverse fungal community. (A) Venn diagram depicting the number of ASVs and the percentage of shared fungal ASVs between compartments sampled from NK (-P) fields in 2019, 2020, and 2021. ASV counts are indicated by the scale on the right. (B) PCoA illustrating the Bray–Curtis dissimilarity of the fungal community compositions of hyphoplane (HP), hyphosphere (HS) at hyphal container position 1, and bulk soil (BS) from NK (-P) fields in 2019, 2020, and 2021. Relative abundance of fungal (sub)phyla (C) and genera (D) in HP, HS, and BS samples, respectively. *n*=18. Created in BioRender. Bucher, M. (2025) https://BioRender.com/ifzbck3.

Next, the relationship between HLD, measured after hyphal extraction using the SSC method, and fungal structures within maize wild-type roots was examined. Interestingly, HLD showed the strongest positive correlation (*R*=0.5–0.8) with the combined presence of intraradical hyphae and arbuscules within *Z. mays* root cells, followed by a slightly weaker correlation with total arbuscular mycorrhizal root colonization which encompasses two types of colonization: roots containing hyphae and arbuscules, and roots containing hyphae, arbuscules, and vesicles. This suggests that extraradical hyphal growth is closely linked to stages of active arbuscular mycorrhizal symbiosis (Fig. 4). Since AMF are not the dominant fungal subphylum in the hyphoplane samples and do not account for all extraradical hyphae extracted using the SSC method, these findings further suggest that active arbuscular mycorrhizal symbiosis influences the growth of a broader community of filamentous fungi (Fig. 5C). This points to a potential, previously unrecognized, feedback mechanism between intraradical colonization and extraradical hyphal growth. Supporting this, Camenzind and Rillig (2013) found that the total amount of AMF and non-AMF hyphae was significantly correlated.

To date, only a few studies have demonstrated a strong positive correlation (*R*≤0.96) between root-internal mycorrhization and extraradical hyphal mass (Camenzind and Rillig, 2013; Qin *et al*., 2022). In contrast, several reports indicate that this relationship is not always consistent. For example, a study of 30 maize lines found a non-significant and even slightly negative correlation between root colonization and ERH development (Sawers *et al*., 2017). Similarly, van Aarle *et al*. (2002), using a mesh bag system with *Plantago lanceolata* grown in sterile sand and inoculated with either *Scutellospora calospora* or *R. irregularis* (syn. *Glomus intraradices*), reported no clear relationship between extraradical mycelium in the hyphal compartment and the extent of root colonization. Collectively, these findings highlight that the correlation between internal colonization and ERH growth can vary widely depending on fungal species, host genotype, substrate, and experimental design.

Nevertheless, the correlation strength observed here (*R*=0.5–0.8), despite reflecting hyphal length from a diverse fungal community, is consistent with findings from natural ecosystems where measurements focus specifically on AMF. For example, Barceló *et al*. (2020) demonstrated a significant positive relationship between the total length of AMF-colonized roots and soil AMF abundance, as indicated by neutral lipid fatty acid (NLFA) 16:1ω5 content (*R*=0.74, *P*<0.001), a biomarker exclusive to AMF. Their study further showed that root length colonized by arbuscules correlated most strongly with soil mycelial biomass, followed by abundance of hyphae and vesicles in colonized roots. This mirrors our observation that HLD is most tightly associated with the presence of intraradical hyphae and arbuscules, reinforcing the idea that ERH development is most robust during metabolically active phases of the symbiosis.

However, at position 3 of the hyphal container, located in the topsoil and furthest from the root system center, this correlation was absent. We can currently only speculate that the lack of correlation at this most distant topsoil position may result from reduced nutrient transfer to the plant host and/or carbon transfer from host to fungus with increasing distance (Jansa *et al*., 2003), potentially disrupting a feedback loop between intraradical and extraradical fungal hyphae. This effect might be further influenced by local soil nutrient status. A follow-up study will provide deeper insights into the relationship between extraradical hyphal length, soil and plant nutrient availability, and the composition of hyphosphere/hyphoplane communities.

#### Recovery of fungi from the hyphoplane

The SSC approach effectively enriched fungal hyphae from a highly diverse community sampled across three consecutive field experiments, with the phylum Ascomycota and the genera *Minimedusa* and *Phialophora* dominating the hyphoplane (Fig. 5C, D). These results corroborate previous findings that Ascomycota are abundant in hyphoplane samples, where hyphae were sampled from a sand–soil mixture using wet-sieving and manual picking with a stereomicroscope, and are similarly abundant in bulk soil and the root endosphere (Zhang *et al*., 2024).

Within the hyphoplane-associated fungal community, we detected sequences affiliated with the genus *Minimedusa*, a group of cellulolytic, saprotrophic fungi in the order Cantharellales. The species *Minimedusa polymorpha* has been described as functionally important, capable of degrading complex carbohydrates and accumulating key biogenic elements such as nitrogen (N), phosphorus (P), sulfur (S), potassium (K), and calcium (Ca) (Pinzari *et al*., 2014). Members of this genus have been found in the rhizosphere and root endosphere of maize under long-term monoculture, where they may help alleviate macronutrient depletion (Wachowska and Rychcik, 2023). To our knowledge, *Minimedusa* has not previously been reported in hyphosphere or hyphoplane samples from maize fields. This may be due to methodological constraints in earlier work or the general lack of genus-level resolution in studies of hyphae-associated compartments. The detection of *Minimedusa* in our study may reflect the improved resolution provided by the SSC technique.

Fungi of this genus are well adapted to low-N conditions, in contrast to AMF, which typically require higher N availability. This difference may allow both groups to occupy similar ecological niches without direct competition (Pinzari *et al*., 2014; Hodge and Storer, 2015). In addition, *Minimedusa* species have been associated with inorganic P solubilization, suggesting that they could contribute to P availability at the hyphoplane and indirectly support plant growth (Ceci *et al*., 2018; Větrovský *et al*., 2020; Spinelli *et al*., 2022).

In our study, sequences affiliated with the genus *Phialophora* were detected in hyphoplane samples, suggesting their potential association with extraradical hyphae. Several strains of this genus have been identified in soil environments and are known to have a global distribution (Li *et al*., 2017). However, their presence in the hyphosphere or hyphoplane has not yet been explicitly documented. Further studies are needed to elucidate the ecological dynamics of *Phialophora* species in the hyphoplane and their potential interactions with other microorganisms.

It has not escaped our attention that the mechanical sieving using a 40 µm mesh of the SSC method might favor the extraction of thicker hyphae over finer ones or those embedded in dense hyphal networks. Indeed, Basidiomycota, which typically form medium to thick hyphae (∼4–8 µm in diameter; Sundari *et al*., 2018), were relatively more abundant in hyphoplane samples compared with the unsieved hyphosphere and bulk soil samples, suggesting a mechanical enrichment of thicker hyphae by SSC. In parallel, Ascomycota, often characterized by thinner hyphae (2–5 µm; Cousin, 2014), showed a lower relative abundance in the hyphoplane, suggesting a reduced recovery of finer hyphae by the SSC method. Supporting the idea of selective loss of finer hyphae during SSC, Mortierellomycotina, a subphylum with generally thin hyphae (Fisher and Roberson, 2016), were more abundant in the hyphosphere and bulk soil compared with the sieved hyphoplane samples. A similar pattern was observed for Mucoromycota, which were also under-represented in the hyphoplane. This group includes Mucoromycotina fine root endophytes (MFRE), known for their very fine hyphae (<2 µm; Thippayarugs *et al*., 1999; Orchard *et al*., 2017), although some Mucoromycota species can form hyphae up to 16 µm in diameter (Alexopoulos *et al*., 1996). However, this interpretation is complicated by the fact that SSC also captures fungal spores. Some spores, especially among Ascomycota, can reach several hundred micrometers or more (Alexopoulos *et al*., 1996), potentially introducing a taxonomic bias not related to hyphal diameter. As a result, increased representation of a given phylum in the SSC fraction may reflect spore enrichment rather than hyphal properties.

Taken together, relative abundance data alone are insufficient to conclusively demonstrate a size-based recovery bias. Nonetheless, the phylum-level patterns are compatible with the idea that SSC, like all sieve-based methods, preferentially retains thicker hyphae, although fine hyphal species remain detectable in hyphoplane samples. Additionally, differences in DNA extraction efficiency across fungal and bacterial taxa may affect sequencing-based community profiles.

The SCC method does not selectively enrich AMF hyphae with attached microbes from field soil, as similarly sized hyphae from other fungal subphyla are also retained. As a result, identifying bacteria specifically associated with AMF hyphae and their role in nutrient cycling remains challenging. Although it is theoretically possible to exclude non-mycorrhizal fungi by examining hyphae morphologically, such procedures (e.g. staining and microscopic selection) may compromise DNA recovery and potentially alter the microbial community. Therefore, our approach is designed to characterize the microbial community associated with hyphae across multiple fungal subphyla, while preserving nucleic acid integrity for subsequent molecular analyses. To ensure high DNA quality and minimize changes in the microbial community during sample processing, the SSC method was performed at a maximum of 4 °C; whether this approach also maintains sufficient RNA quality remains to be determined.

#### Recovery of bacteria from the hyphoplane

**Fig. 6.**
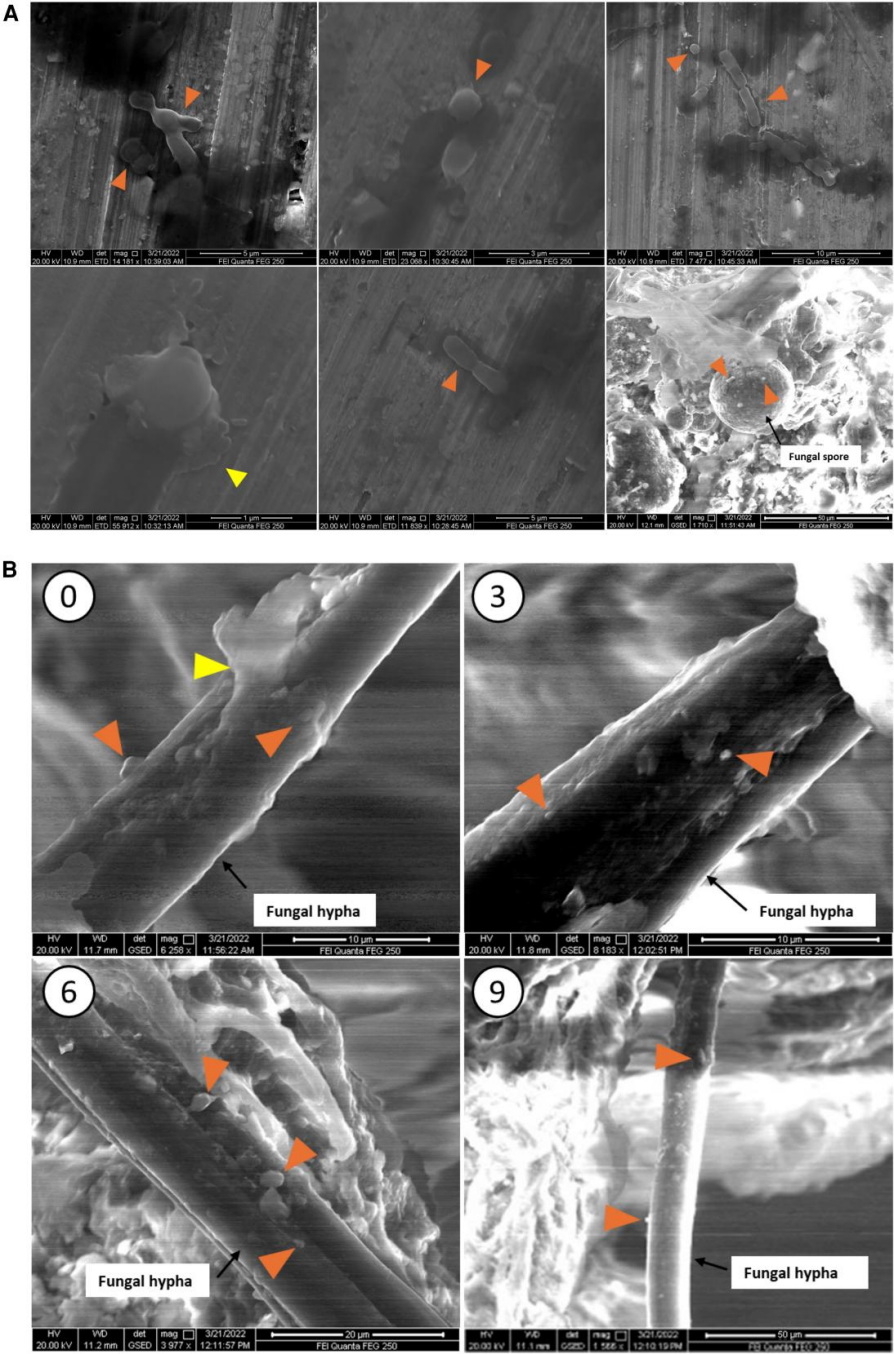
SEM of microbial attachment to fungal hyphae after each critical step of the SSC method. (A) SEM images taken of bacteria (control samples). (B) 0, 3, 6, and 9 indicate the steps of the SSC method before starting the method, after the second sieving step, after the last washing step, and after freeze-drying. Orange arrowheads indicate bacteria, and yellow arrowheads show the bacterial biofilm. Created in BioRender. Bucher, M. (2025) https://BioRender.com/lwcurp2.

Importantly, SEM, PCR-based detection, and DAPI staining confirmed that bacteria remained attached to fungal hyphae after extraction from field soil using the SSC method (Fig. 6; Supplementary Fig. S5), demonstrating that SSC is well suited for studying bacterial hyphoplane communities in field experiments without the need for additives in natural substrates. However, while SEM confirms bacterial attachment, it does not quantify the proportion of hyphae colonized or the density of bacterial association.

**Fig. 7.**
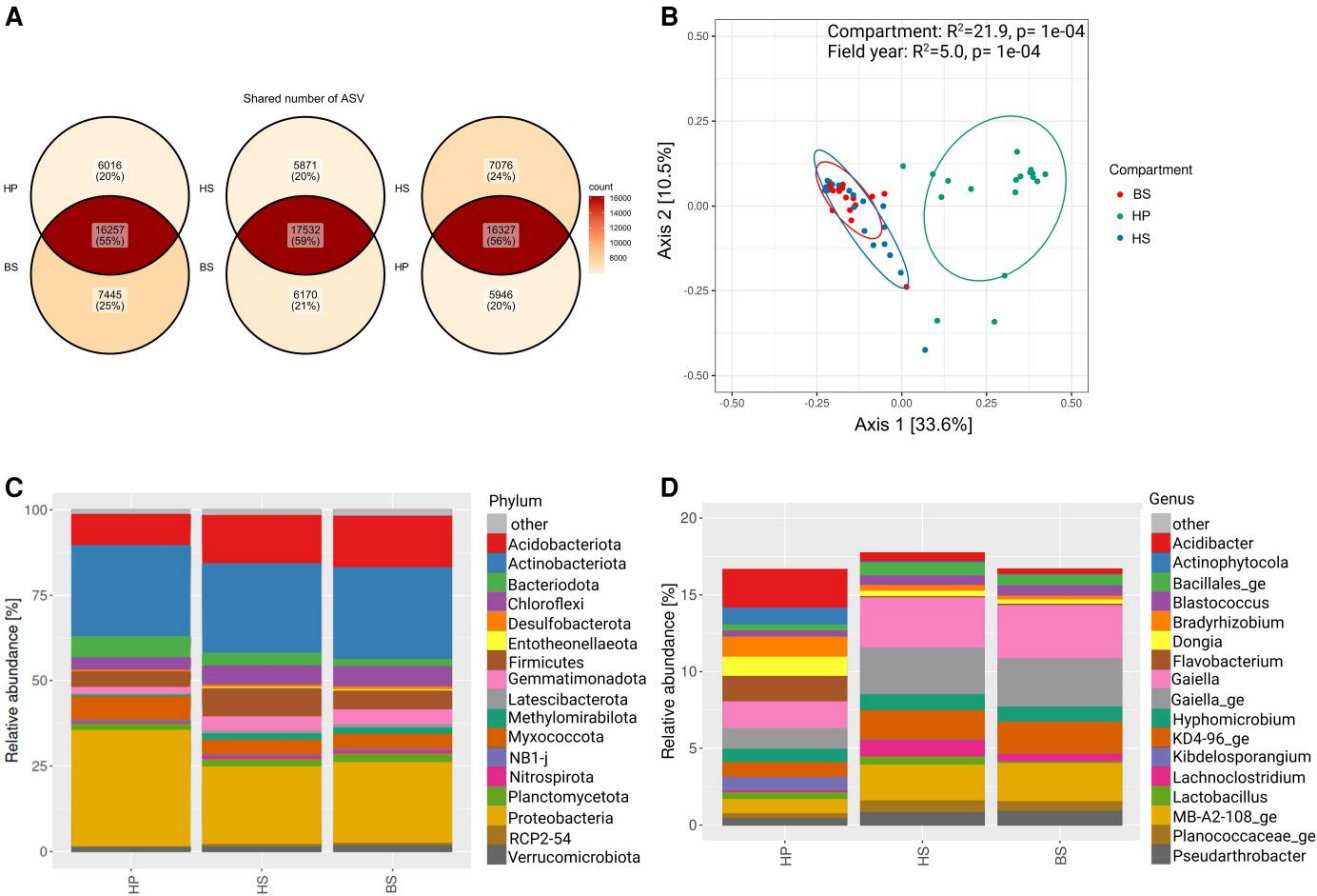
Bacterial community composition differs between hyphoplane and soil compartments. (A) Venn diagram depicting the number of ASVs and the percentage of shared bacterial ASVs between compartments sampled from NK (-P) fields in 2019, 2020, and 2021. The color scale represents the number of ASV counts, ranging from bright orange (low number of ASVs) to dark red (high number of ASVs). (B) PCoA illustrates the Bray–Curtis dissimilarity of the bacterial community compositions of hyphoplane (HP, green) and hyphosphere (HS, blue) at hyphal container position 1, and bulk soil (BS, red) from NK (-P) fields in 2019, 2020, and 2021. Relative abundance of bacterial phyla (C) and genera (D) in HP, HS, and BS samples, respectively. *n*=18. Created in BioRender. Bucher, M. (2025) https://BioRender.com/rhi9hmu.

The bacterial hyphoplane community was dominated by the phyla Proteobacteria and Actinobacteriota (Fig. 7C), consistent with findings from previous hyphoplane studies (Emmett *et al*., 2021; Wang *et al*., 2022; Zhang *et al*., 2024). Within this community, the genera *Acidibacter* and *Flavobacterium* were the most abundant taxa (Fig. 7D). In line with our findings, *Acidibacter* were previously reported to associate with *Glomus versiforme* hyphae harvested from a mesocosm comprising a plant compartment sown with two *Brachypodium distachyon* seeds and hyphal in-growth cores filled with soil (Emmett *et al*., 2021).

Although, to our knowledge, previous studies have not explicitly quantified the abundance of *Flavobacterium* in the hyphosphere or on the hyphoplane, it is well established that members of the phylum Bacteroidota, including *Flavobacterium*, are common in the rhizosphere and root endosphere, where the latter can reach relative abundances of ∼5% to >20% (Xu *et al*., 2018; Seo *et al*., 2024). Species of the genus *Flavobacterium* are classified as plant growth-promoting rhizobacteria, enhancing plant growth in the rhizosphere and rhizoplane by degrading complex organic compounds, such as carbohydrates and amino acids, exuded by roots and arbuscular mycorrhizal hyphae (Bernardet and Nakagawa, 2006; Bulgarelli *et al*., 2012; Kolton *et al*., 2016; Wang *et al*., 2021). It is important to note that these communities may also include microbes residing within the hyphal endosphere, although they are likely to be a minority, as only a few specific taxa have been identified as hyphal endosymbionts. For example, *Candidatus Glomeribacter gigasporarum*, a rod-shaped, Gram-negative beta-proteobacterium, has been consistently associated with Glomeromycotina (Bonfante *et al*., 1994; Salvioli *et al*., 2016). Additionally, Glomeromycotina host round-shaped bacteria resembling Gram-positive bacteria, known as Mollicutes-related endobacteria (Naumann *et al*., 2010). Future studies will enable a clearer experimental distinction between bacteria inhabiting the hyphal endosphere and those colonizing the hyphoplane, providing deeper insights into their ecological roles.

#### Comparison of microbial communities in hyphoplane, hyphosphere, and bulk soil

Our findings show that 22% of fungal ASVs were shared between the hyphoplane and bulk soil, suggesting that these fungi probably exhibit filamentous growth (Fig. 5A). This also indicates that the SSC method successfully enriched 22% of the total soil fungal diversity by specifically targeting filamentous fungi. Fungal community composition in the hyphosphere has received limited attention, as most existing studies primarily focus on bacterial communities. Among the few that do address fungal taxa, comparative analyses of ASVs between bulk soil and the hyphosphere are generally lacking, which limits the availability of data directly comparable with our study (Xu *et al*., 2018).

For bacteria, 55% of the taxa detected in bulk soil were also found in the hyphoplane, while this proportion was 4% higher in the hyphosphere (Fig. 7A). The overlap of ASVs between the hyphosphere and hyphoplane was 1–2% lower than that between the hyphoplane and bulk soil. A comparative study reported that between 12% and 82% of ASVs were shared between bulk soil and the hyphoplane, depending on the experimental set-up, which varied between compartments with different substrates and those with homogeneous substrates (Zhang *et al*., 2024). This supports that our methodology captured a sufficiently representative portion of the hyphoplane-associated community. Differences between our results and those of Zhang *et al*. may also be explained by the use of natural soil without added sand in our study, as well as by conducting experiments under field conditions, which are more ecologically relevant but less controlled (Zhang *et al*., 2024). Finally, the sieving and centrifugation steps in SSC probably remove loosely attached microorganisms, thus enriching for those tightly associated with the hyphal surface or embedded in biofilms, as illustrated in Fig. 6.

To further assess differences in fungal and bacterial community composition across compartments, beta diversity was analyzed. PCoA revealed that fungal and bacterial communities in the hyphoplane were clearly distinct from those in bulk soil and the hyphosphere (Figs 5B, 7B), confirming earlier findings of distinct hyphoplane microbiota (Gahan and Schmalenberger, 2015; Zhang *et al*., 2018, 2024; Wang *et al*., 2019; Zhou *et al*., 2020; Emmett *et al*., 2021; Wang *et al*., 2022). In contrast, bulk soil and hyphosphere communities exhibited substantial overlap, suggesting that conditions within the container closely resemble those of natural soil environments. PERMANOVA confirmed that beta diversity was primarily affected by compartment type (31.0% and 21.9% of the variance for fungi and bacteria, respectively) and less by the year in which the experiment was conducted (6.0% and 5.0%), suggesting consistent patterns across field years.

## Conclusion

In conclusion, this study presents a method that improves upon previous protocols by combining, optimizing, and automating the wet-sieving steps, enabling a 2- to 2.5-fold faster and more efficient extraction of hyphae from natural substrates while reducing operator-dependent variability compared with earlier approaches. Unlike earlier approaches, this method avoids sand or glass bead supplementation, preserving the natural plant–soil–microbe continuum. By streamlining these processes, this methodology not only facilitates efficient hyphae extraction, but also preserves and enables the targeted study of microbes specifically attached to fungal hyphae in large-scale field studies. This provides a valuable tool to investigate how soil management and plant genotype affect the composition and function of hyphoplane microbial communities.

## Supplementary Material

eraf539_Supplementary_Data

## Data Availability

The description of the sieving and sucrose centrifugation method is openly available on protocols.io at https://doi.org/10.17504/protocols.io.x54v95p8pl3e/v1.

## Supplementary data

The following supplementary data are available at *JXB* online.

Fig. S1. Effects of sieving amplitude, water pressure, and medium gravel during the wet sieving step of the SSC method were tested to determine optimal parameters.

Fig. S2. The amount of residual material after processing using the SSC method was evaluated for different soil types, with sandy soils showing higher residues than clay-rich soils.

Fig. S3. Bacterial contamination on sieves after fungal hyphae extraction was lowest following sonication and water washing, as determined by PCR targeting the bacterial 16S rRNA gene, compared with UV light or hypochlorite treatments.

Fig. S4. A comparison of methods for quantifying hyphal density in full, half, and quarter soil sample weights showed that automated image analysis (AnaMorf and HyLength) overestimated the values compared with manual VGI counts.

Fig. S5. SEM, PCR-based detection, and DAPI staining confirmed that bacteria remained attached to fungal hyphae after extraction from field soil using the SSC method.

## Abbreviations

AMF: arbuscular mycorrhizal fungi
BSM: aqueous blending and sieving method
ERH: extraradical hyphae
HC: hyphal container
HLD: hyphal length density
MAE: mean absolute error
MPE: mean percentage error
NGS: next-generation sequencing
PCoA: principal coordinates analysis
SSC: sieving and sucrose centrifugation
VGI: visual gridline intersection
WSCM: differential water/sucrose centrifugation method

## References

[eraf539-B1] Abbott LK, Robson AD, De Boer G. 1984. The effect of phosphorus on the formation of hyphae in soil by the vesicular-arbuscular mycorrhizal fungus, *Glomus fasciculatum*. New Phytologist 97, 437–446.

[eraf539-B2] Abarenkov K, Nilsson RH, Larsson KH, et al 2024. The UNITE database for molecular identification and taxonomic communication of fungi and other eukaryotes: sequences, taxa and classifications reconsidered. Nucleic Acids Research 52, D791–D797. 37953409 10.1093/nar/gkad1039 PMC10767974

[eraf539-B3] Alexopoulos CJ, Mims CW, Blackwell M. 1996. Introductory mycology. New York: Wiley & Sons.

[eraf539-B4] Allen MF, Moore TS, Christensen M, Stanton N. 1979. Growth of vesicular-arbuscular-mycorrhizal and nonmycorrhizal *Bouteloua gracilis* in a defined medium. Mycologia 71, 666–669.

[eraf539-B5] Awad A, Pena R. 2023. An improved method for extraction of soil fungal mycelium. MethodsX 11, 102477. 38023315 10.1016/j.mex.2023.102477 PMC10679939

[eraf539-B6] Barceló M, van Bodegom PM, Tedersoo L, den Haan N, Ciska Veen GF, Ostonen I, Trimbos K, Soudzilovskaia NA. 2020. The abundance of arbuscular mycorrhiza in soils is linked to the total length of roots colonized at ecosystem level. PLoS One 15, e0237256. 32915795 10.1371/journal.pone.0237256 PMC7485760

[eraf539-B7] Barry DJ, Chan C, Williams GA. 2009. Morphological quantification of filamentous fungal development using membrane immobilization and automatic image analysis. Journal of Industrial Microbiology & Biotechnology 36, 787–800. 19277741 10.1007/s10295-009-0552-9

[eraf539-B8] Barry DJ, Williams GA, Chan C. 2015. Automated analysis of filamentous microbial morphology with AnaMorf. Biotechnology Progress 31, 849–852. 25864556 10.1002/btpr.2087

[eraf539-B9] Bauer DF . 1972. Constructing confidence sets using rank statistics. Journal of the American Statistical Association 67, 687–690.

[eraf539-B10] Bernardet JF, Nakagawa Y. 2006. An introduction to the family Flavobacteriaceae. In: Dworkin M, Falkow S, Rosenberg E, Schleifer KH, Stackebrandt E, eds. The prokaryotes. New York: Springer, 455–480.

[eraf539-B11] Bingle WH, Paul EA. 1986. A method for separating fungal hyphae from soil. Canadian Journal of Microbiology 32, 62–66.

[eraf539-B12] Bonfante P, Balestrini R, Gen KM. 1994. Storage and secretion processes in the spore of *Gigaspora margarita* Becker & Hall as revealed by high-pressure freezing and freeze substitution. New Phytologist 128, 93–101. 33874525 10.1111/j.1469-8137.1994.tb03991.x

[eraf539-B13] Boyce KJ, Andrianopoulos A. 2015. Fungal dimorphism: the switch from hyphae to yeast is a specialized morphogenetic adaptation allowing colonization of a host. FEMS Microbiology Reviews 39, 797–811. 26253139 10.1093/femsre/fuv035

[eraf539-B14] Boyno G, Demir S, Danesh YR, Durak ED, Çevik R, Farda B, Djebaili R, Pellegrini M. 2023. A new technique for the extraction of arbuscular mycorrhizae fungal spores from rhizosphere. Journal of Fungi (Basel) 9, 845. 10.3390/jof9080845 PMC10455966 37623616

[eraf539-B15] Bulgarelli D, Rott M, Schlaeppi K, et al 2012. Revealing structure and assembly cues for *Arabidopsis* root-inhabiting bacterial microbiota. Nature 488, 91–95. 22859207 10.1038/nature11336

[eraf539-B16] Callahan BJ, McMurdie PJ, Rosen MJ, Han AW, Johnson AJA, Holmes SP. 2016. DADA2: high-resolution sample inference from Illumina amplicon data. Nature Methods 13, 581–583. 27214047 10.1038/nmeth.3869 PMC4927377

[eraf539-B17] Cambardella CA, Elliott ET. 1992. Particulate soil organic-matter changes across a grassland cultivation sequence. Soil Science Society of America Journal 56, 777–783.

[eraf539-B18] Camenzind T, Rillig MC. 2013. Extraradical arbuscular mycorrhizal fungal hyphae in an organic tropical montane forest soil. Soil Biology & Biochemistry 64, 96–102.

[eraf539-B19] Caporaso JG, Lauber CL, Walters WA, Berg-Lyons D, Lozupone CA, Turnbaugh PJ, Fierer N, Knight R. 2011. Global patterns of 16S rRNA diversity at a depth of millions of sequences per sample. Proceedings of the National Academy of Sciences, USA 108, 4516–4522. 10.1073/pnas.1000080107 PMC3063599 20534432

[eraf539-B20] Cardini A, Pellegrino E, Del Dottore E, Gamper HA, Mazzolai B, Ercoli L. 2020. Hylength: a semi-automated digital image analysis tool for measuring the length of roots and fungal hyphae of dense mycelia. Mycorrhiza 30, 229–242. 32300867 10.1007/s00572-020-00956-w

[eraf539-B21] Ceci A, Pinzari F, Russo F, Maggi O, Persiani AM. 2018. Saprotrophic soil fungi to improve phosphorus solubilisation and release: *in vitro* abilities of several species. Ambio 47, 30–40. 29159452 10.1007/s13280-017-0972-0 PMC5722741

[eraf539-B22] Coleman AW . 1980. Enhanced detection of bacteria in natural environments by fluorochrome staining of DNA. Limnology and Oceanography 25, 948–951.

[eraf539-B23] Cousin MA . 2014. FUNGI | classification of the eukaryotic ascomycetes. In: Batt CA, Tortorello ML, eds. Encyclopedia of food microbiology, 2nd edn. Academic Press, 35–40.

[eraf539-B24] de Mendiburu MF . 2019. Package ‘agricolae.’ R package version 1-2. http://CRAN.R-project.org/package=agricolae

[eraf539-B25] Dray S, Dufour A-B. 2007. The ade4 package: implementing the duality diagram for ecologists. Journal of Statistical Software 22, 1–20.

[eraf539-B26] Emmett BD, Lévesque-Tremblay V, Harrison MJ. 2021. Conserved and reproducible bacterial communities associate with extraradical hyphae of arbuscular mycorrhizal fungi. The ISME Journal 15, 2276–2288. 33649552 10.1038/s41396-021-00920-2 PMC8319317

[eraf539-B27] Fisher KE, Roberson RW. 2016. Hyphal tip cytoplasmic organization in four zygomycetous fungi. Mycologia 108, 533–542. 26908648 10.3852/15-226

[eraf539-B28] Gahan J, Schmalenberger A. 2015. Arbuscular mycorrhizal hyphae in grassland select for a diverse and abundant hyphospheric bacterial community involved in sulfonate desulfurization. Applied Soil Ecology 89, 113–121.

[eraf539-B29] Ganther M, Lippold E, Bienert MD, Bouffaud M-L, Bauer M, Baumann L, Bienert GP, Vetterlein D, Heintz-Buschart A, Tarkka MT. 2022. Plant age and soil texture rather than the presence of root hairs cause differences in maize resource allocation and root gene expression in the field. Plants (Basel) 11, 2883. 36365336 10.3390/plants11212883 PMC9657941

[eraf539-B30] Hanssen JF, Thingstad TF, Goksøyr J. 1974. Evaluation of hyphal lengths and fungal biomass in soil by a membrane filter technique. Oikos 25, 102–107.

[eraf539-B31] Hodge A, Storer K. 2015. Arbuscular mycorrhiza and nitrogen: implications for individual plants through to ecosystems. Plant and Soil 386, 1–19.

[eraf539-B32] Hollander M, Wolfe DA. 1973. Nonparametric statistical methods. Hoboken, NJ: John Wiley & Sons, 115–120.

[eraf539-B33] Jakobsen I, Rosendahl L. 1990. Carbon flow into soil and external hyphae from roots of mycorrhizal cucumber plants. New Phytologist 115, 77–83.

[eraf539-B34] Jakobsen I, Abbott LK, Robson AD. 1992. External hyphae of vesicular-arbuscular mycorrhizal fungi associated with *Trifolium subterraneum* L. New Phytologist 120, 371–380.

[eraf539-B35] Jansa J, Mozafar A, Frossard E. 2003. Long-distance transport of P and Zn through the hyphae of an arbuscular mycorrhizal fungus in symbiosis with maize. Agronomie 23, 481–488.

[eraf539-B36] Jiang F, Zhang L, Zhou J, George TS, Feng G. 2021. Arbuscular mycorrhizal fungi enhance mineralisation of organic phosphorus by carrying bacteria along their extraradical hyphae. New Phytologist 230, 304–315. 33205416 10.1111/nph.17081

[eraf539-B37] Jiang Y, Wang W, Xie Q, et al 2017. Plants transfer lipids to sustain colonization by mutualistic mycorrhizal and parasitic fungi. Science 356, 1172–1175. 28596307 10.1126/science.aam9970

[eraf539-B38] Johnson SR, Martin DH, Cammarata C, Morse SA. 1995. Alterations in sample preparation increase sensitivity of PCR assay for diagnosis of chancroid. Journal of Clinical Microbiology 33, 1036–1038. 7790433 10.1128/jcm.33.4.1036-1038.1995 PMC228097

[eraf539-B39] Jones PCT, Mollison JE, Quenouille MH. 1948. A technique for the quantitative estimation of soil micro-organisms. Microbiology 2, 54–69. 10.1099/00221287-6-3-4-261 14927873

[eraf539-B40] Keymer A, Pimprikar P, Wewer V, et al 2017. Lipid transfer from plants to arbuscular mycorrhiza fungi. eLife 6, e29107. 28726631 10.7554/eLife.29107 PMC5559270

[eraf539-B41] Kolton M, Erlacher A, Berg G, Cytryn, E. 2016. The *Flavobacterium* genus in the plant holobiont: ecological, physiological, and applicative insights. In: Castro-Sowinski S, ed. Microbial models: from environmental to industrial sustainability. Microorganisms for sustainability, Vol 1. Singapore: Springer, 189–207.

[eraf539-B42] Li X-L, Marschner H, George E. 1991. Acquisition of phosphorus and copper by VA-mycorrhizal hyphae and root-to-shoot transport in white clover. Plant and Soil 136, 49–57.

[eraf539-B43] Li Y, Xiao J, de Hoog GS, Wang X, Wan Z, Yu J, Liu W, Li R. 2017. Biodiversity and human-pathogenicity of *Phialophora verrucosa* and relatives in Chaetothyriales. Persoonia 38, 1–19. 29151624 10.3767/003158517X692779 PMC5645179

[eraf539-B44] Long T, Or D. 2007. Microbial growth on partially saturated rough surfaces: simulations in idealized roughness networks. Water Resources Research 43, W02409.

[eraf539-B45] McMurdie PJ, Holmes S. 2013. Phyloseq: an R package for reproducible interactive analysis and graphics of microbiome census data. PLoS One 8, e61217. 23630581 10.1371/journal.pone.0061217 PMC3632530

[eraf539-B46] Nagy R, Karandashov V, Chague V, Kalinkevich K, Tamasloukht M, Xu G, Jakobsen I, Levy AA, Amrhein N, Bucher M. 2005. The characterization of novel mycorrhiza-specific phosphate transporters from *Lycopersicon esculentum* and *Solanum tuberosum* uncovers functional redundancy in symbiotic phosphate transport in solanaceous species. The Plant Journal 42, 236–250. 15807785 10.1111/j.1365-313X.2005.02364.x

[eraf539-B47] Naumann M, Schussler A, Bonfante P. 2010. The obligate endobacteria of arbuscular mycorrhizal fungi are ancient heritable components related to the Mollicutes. The ISME Journal 4, 862–871. 20237515 10.1038/ismej.2010.21

[eraf539-B48] Newman EI . 1966. A method of estimating the total length of root in a sample. The Journal of Applied Ecology 3, 139–145.

[eraf539-B49] Nuccio EE, Hodge A, Pett-Ridge J, Herman DJ, Weber PK, Firestone MK. 2013. An arbuscular mycorrhizal fungus significantly modifies the soil bacterial community and nitrogen cycling during litter decomposition. Environmental Microbiology 15, 1870–1881. 23360621 10.1111/1462-2920.12081

[eraf539-B50] Oksanen J, Blanchet FG, Kindt R, Legendre P, O'Hara RG, Simpson GL, Minchin P, O’Hara RB. 2007. Vegan: community ecology package. R package version 1.8-5. https://vegandevs.github.io/vegan/

[eraf539-B51] Orchard S, Hilton S, Bending GD, et al 2017. Fine endophytes (*Glomus tenue*) are related to Mucoromycotina, not Glomeromycota. New Phytologist 213, 481–486. 27768808 10.1111/nph.14268

[eraf539-B52] Pfeffer PE, Douds Jr DD, Becard G, Shachar-Hill Y. 1999. Carbon uptake and the metabolism and transport of lipids in an arbuscular mycorrhiza. Plant Physiology 120, 587–598. 10364411 10.1104/pp.120.2.587 PMC59298

[eraf539-B53] Pinzari F, Reverberi M, Piñar G, Maggi O, Persiani AM. 2014. Metabolic profiling of *Minimedusa polyspora* (Hotson) Weresub & P.M. LeClair, a cellulolytic fungus isolated from Mediterranean maquis, in southern Italy. Plant Biosystems 148, 333–341.

[eraf539-B54] Prout JN, Williams A, Wanke A, Schornack S, Ton J, Field KJ. 2024. Mucoromycotina ‘fine root endophytes’: a new molecular model for plant–fungal mutualisms? Trends in Plant Science 29, 650–661. 38102045 10.1016/j.tplants.2023.11.014

[eraf539-B55] Qin Y, Zhang W, Feng Z, Feng G, Zhu H, Yao Q. 2022. Arbuscular mycorrhizal fungus differentially regulates P mobilizing bacterial community and abundance in rhizosphere and hyphosphere. Applied Soil Ecology 170: 104294.

[eraf539-B56] Quast C, Pruesse E, Yilmaz P, Gerken J, Schweer T, Yarza P, Peplies J, Glöckner FO. 2013. The SILVA ribosomal RNA gene database project: improved data processing and web-based tools. Nucleic Acids Research 41, D590–D596. 23193283 10.1093/nar/gks1219 PMC3531112

[eraf539-B57] Richter F, Calonne-Salmon M, van der Heijden MGA, Declerck S, Stanley CE. 2024. AMF-SporeChip provides new insights into arbuscular mycorrhizal fungal asymbiotic hyphal growth dynamics at the cellular level. Lab on a Chip 24, 1930–1946. 38416560 10.1039/d3lc00859b PMC10964749

[eraf539-B58] Royston JP . 1982. An extension of Shapiro and Wilk’s W test for normality to large samples. Applied Statistics 31, 115–124.

[eraf539-B59] Royston P . 1995. Remark AS R94: a remark on algorithm AS 181: the W-test for normality. Applied Statistics 44, 547–551.

[eraf539-B60] Salvioli A, Ghignone S, Novero M, Navazio L, Venice F, Bagnaresi P, Bonfante P. 2016. Symbiosis with an endobacterium increases the fitness of a mycorrhizal fungus, raising its bioenergetic potential. The ISME Journal 10, 130–144. 26046255 10.1038/ismej.2015.91 PMC4681866

[eraf539-B61] Sawers RJH, Svane SF, Quan C, et al 2017. Phosphorus acquisition efficiency in arbuscular mycorrhizal maize is correlated with the abundance of root-external hyphae and the accumulation of transcripts encoding PHT1 phosphate transporters. New Phytologist 214, 632–643. 28098948 10.1111/nph.14403

[eraf539-B62] Scheublin TR, Sanders IR, Keel C, van der Meer JR. 2010. Characterisation of microbial communities colonising the hyphal surfaces of arbuscular mycorrhizal fungi. The ISME Journal 4, 752–763. 20147983 10.1038/ismej.2010.5

[eraf539-B63] Schindelin J, Arganda-Carreras I, Frise E, et al 2012. Fiji: an open-source platform for biological-image analysis. Nature Methods 9, 676–682. 22743772 10.1038/nmeth.2019 PMC3855844

[eraf539-B64] Seo H, Kim JH, Lee S-M, Lee S-W. 2024. The plant-associated Flavobacterium: a hidden helper for improving plant health. Plant Pathology Journal 40, 251–260. 38835296 10.5423/PPJ.RW.01.2024.0019 PMC11162857

[eraf539-B65] Shen Q, Kirschbaum MU, Hedley MJ, Camps Arbestain M. 2016. Testing an alternative method for estimating the length of fungal hyphae using photomicrography and image processing. PLoS One 11, e0157017. 27284995 10.1371/journal.pone.0157017 PMC4902305

[eraf539-B66] Singara Charya MA . 2015. Fungi: an overview. In: Bahadur B, Venkat Rajam M, Sahijram L, Krishnamurthy K, eds. Plant biology and biotechnology. New Delhi: Springer, 197–215.

[eraf539-B67] Soil Survey Staff . 1975. Soil Taxonomy: A basic system of soil classification for making and interpreting soil surveys. Agriculture Handbook No. 436. Soil Conservation Service, U.S. Department of Agriculture, Washington, DC.

[eraf539-B68] Spatafora JW, Chang Y, Benny GL, et al 2016. A phylum-level phylogenetic classification of zygomycete fungi based on genome-scale data. Mycologia 108, 1028–1046. 27738200 10.3852/16-042 PMC6078412

[eraf539-B69] Spinelli V, Brasili E, Sciubba F, Ceci A, Giampaoli O, Miccheli A, Pasqua G, Persiani AM. 2022. Biostimulant effects of *Chaetomium globosum* and *Minimedusa polyspora* culture filtrates on *Cichorium intybus* plant: growth performance and metabolomic traits. Frontiers in Plant Science 13, 879076. 35646045 10.3389/fpls.2022.879076 PMC9134003

[eraf539-B70] Sundari T, Anand AAP, Jenifer P, Shenbagarathai R. 2018. Bioprospection of Basidiomycetes and molecular phylogenetic analysis using internal transcribed spacer (ITS) and 5.8S rRNA gene sequence. Scientific Reports 8, 10720. 30013072 10.1038/s41598-018-29046-w PMC6048145

[eraf539-B71] Taudière A . 2023. MiscMetabar: an R package to facilitate visualization and reproducibility in metabarcoding analysis. Journal of Open Source Software 8, 6038.

[eraf539-B72] Tennant D . 1975. A test of a modified line intersect method of estimating root length. The Journal of Ecology 63, 995–1001.

[eraf539-B73] Thippayarugs S, Bansal M, Abbott LK. 1999. Morphology and infectivity of fine endophyte in a Mediterranean environment. Mycological Research 103, 1369–1379.

[eraf539-B74] Tisserant E, Malbreil M, Kuo A, et al 2013. Genome of an arbuscular mycorrhizal fungus provides insight into the oldest plant symbiosis. Proceedings of the National Academy of Sciences, USA 110, 20117–20122. 10.1073/pnas.1313452110 PMC3864322 24277808

[eraf539-B75] Toljander JF, Lindahl BD, Paul LR, Elfstrand M, Finlay RD. 2007. Influence of arbuscular mycorrhizal mycelial exudates on soil bacterial growth and community structure. FEMS Microbiology Ecology 61, 295–304. 17535297 10.1111/j.1574-6941.2007.00337.x

[eraf539-B76] Trépanier M, Becard G, Moutoglis P, Willemot C, Gagné S, Avis TJ, Rioux J-A. 2005. Dependence of arbuscular-mycorrhizal fungi on their plant host for palmitic acid synthesis. Applied and Environmental Microbiology 71, 5341–5347. 16151123 10.1128/AEM.71.9.5341-5347.2005 PMC1214663

[eraf539-B77] Treseder KK, Lennon JT. 2015. Fungal traits that drive ecosystem dynamics on land. Microbiology and Molecular Biology Reviews 79, 243–262. 25971588 10.1128/MMBR.00001-15 PMC4429240

[eraf539-B78] van Aarle IM, Olsson PA, Söderström B. 2002. Arbuscular mycorrhizal fungi respond to the substrate pH of their extraradical mycelium by altered growth and root colonization. New Phytologist 155, 173–182. 33873298 10.1046/j.1469-8137.2002.00439.x

[eraf539-B79] van der Heijden MGA, Martin FM, Selosse M-A, Sanders IR. 2015. Mycorrhizal ecology and evolution: the past, the present, and the future. New Phytologist 205, 1406–1423. 25639293 10.1111/nph.13288

[eraf539-B80] Větrovský T, Morais D, Kohout P, et al 2020. GlobalFungi, a global database of fungal occurrences from high-throughput-sequencing metabarcoding studies. Scientific Data 7, 228. 32661237 10.1038/s41597-020-0567-7 PMC7359306

[eraf539-B81] Vetterlein D, Carminati A, Kögel-Knabner I, Bienert GP, Smalla K, Oburger E, Schnepf A, Banitz T, Tarkka MT, Schlüter S. 2020. Rhizosphere spatiotemporal organization—a key to rhizosphere functions. Frontiers in Agronomy 2, 8.

[eraf539-B82] Wachowska U, Rychcik B. 2023. Plants control the structure of mycorrhizal and pathogenic fungal communities in soil in a 50-year maize monoculture experiment. Plant and Soil 484, 133–153.

[eraf539-B83] Wang F, Kertesz MA, Feng G. 2019. Phosphorus forms affect the hyphosphere bacterial community involved in soil organic phosphorus turnover. Mycorrhiza 29, 351–362. 31044298 10.1007/s00572-019-00896-0

[eraf539-B84] Wang F, Zhang L, Zhou J, Rengel Z, George TS, Feng G. 2022. Exploring the secrets of hyphosphere of arbuscular mycorrhizal fungi: processes and ecological functions. Plant and Soil 481, 1–22.

[eraf539-B85] Wang M, Sun H, Xu L, Xu Z. 2021. Bacterial diversity in tea plant (*Camellia sinensis*) rhizosphere soil from Qinling Mountains and its relationship with environmental elements. Plant and Soil 460, 403–415.

[eraf539-B86] Wang X-X, Hoffland E, Feng G, Kuyper TW. 2017. Phosphate uptake from phytate due to hyphae-mediated phytase activity by arbuscular mycorrhizal maize. Frontiers in Plant Science 8, 684. 28503185 10.3389/fpls.2017.00684 PMC5408084

[eraf539-B87] White TJ, Bruns TD, Lee SB, Taylor JW. 1990. Amplification and direct sequencing of fungal ribosomal RNA genes for phylogenetics. In: Innis MA, Gelfand DH, Sninsky JJ, White TJ, eds. PCR protocols: a guide to methods and applications. Cambridge, MA: Academic Press, 315–322.

[eraf539-B88] Wickham H . 2016. Ggplot2: elegant graphics for data analysis, 2nd edn. Cham: Springer International Publishing, 203–220.

[eraf539-B89] Xu J, Liu S, Song S, Guo H, Tang J, Yong JWH, Ma Y, Chen X. 2018. Arbuscular mycorrhizal fungi influence decomposition and the associated soil microbial community under different soil phosphorus availability. Soil Biology & Biochemistry 120, 181–190.

[eraf539-B90] Zhang C, van der Heijden MGA, Dodds BK, Nguyen TB, Spooren J, Valzano-Held A, Cosme M, Berendsen RL. 2024. A tripartite bacterial–fungal–plant symbiosis in the mycorrhiza-shaped microbiome drives plant growth and mycorrhization. Microbiome 12, 13. 38243337 10.1186/s40168-023-01726-4 PMC10799531

[eraf539-B91] Zhang L, Fan J, Ding X, He X, Zhang F, Feng G. 2014. Hyphosphere interactions between an arbuscular mycorrhizal fungus and a phosphate solubilizing bacterium promote phytate mineralization in soil. Soil Biology & Biochemistry 74, 177–183.

[eraf539-B92] Zhang L, Shi N, Fan J, Wang F, George TS, Feng G. 2018. Arbuscular mycorrhizal fungi stimulate organic phosphate mobilization associated with changing bacterial community structure under field conditions. Environmental Microbiology 20, 2639–2651. 29901256 10.1111/1462-2920.14289

[eraf539-B93] Zhou J, Chai X, Zhang L, George TS, Wang F, Feng G. 2020. Different arbuscular mycorrhizal fungi cocolonizing on a single plant root system recruit distinct microbiomes. mSystems 5, 6. 10.1128/mSystems.00929-20 PMC7771537 33323417

