# Supplementary material for: Streamlining the isolation of fungal hyphae: a semi-automated approach for soil substrates
Source: J Exp Bot. 2025 Dec 16;77(15):4857–73. doi: 10.1093/jxb/eraf539 (PMC13462798; doi:10.1093/jxb/eraf539)
Supplement: Supplementary file 1 [file eraf539_supplementary_data.pdf]

Supplementary Figures S1-S5:

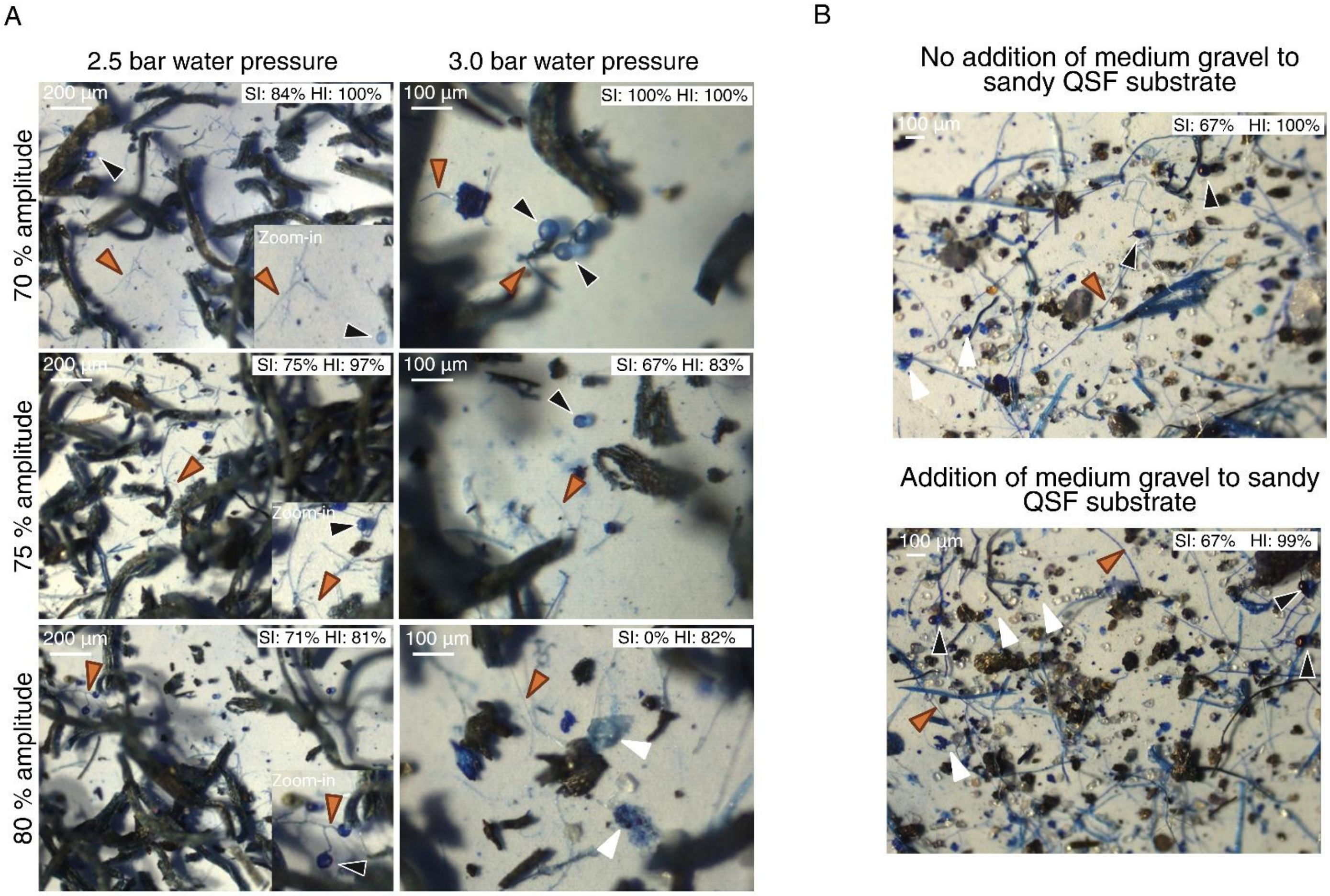

Supplementary Figure S1: Sieving amplitude, water pressure, and the effect of adding medium gravel on hyphal integrity. (A) The sieving machine was set to amplitudes of 40%, 75% and 80%, while the water pressure of the pump was adjusted to either 2.5 or 3.0 bar. Fungal structures were stained with ink solution. The integrity of both hyphae and spores was then analyzed. (B) The effect of adding medium gravel (approximately up to 15 mm in size, not shown) during the sieving process on spore and hyphal integrity within a sandy QSF substrate. Intact hyphae are indicated by orange arrows, intact spores by black arrows, and disrupted spores by white arrows. Integrity was calculated as the number of intact hyphae or spores divided by the total number of hyphae or spores counted, expressed as a percentage. SI = Spore Integrity (% intact spores per total spores); HI = Hyphal Integrity (% intact hyphae per total hyphae).

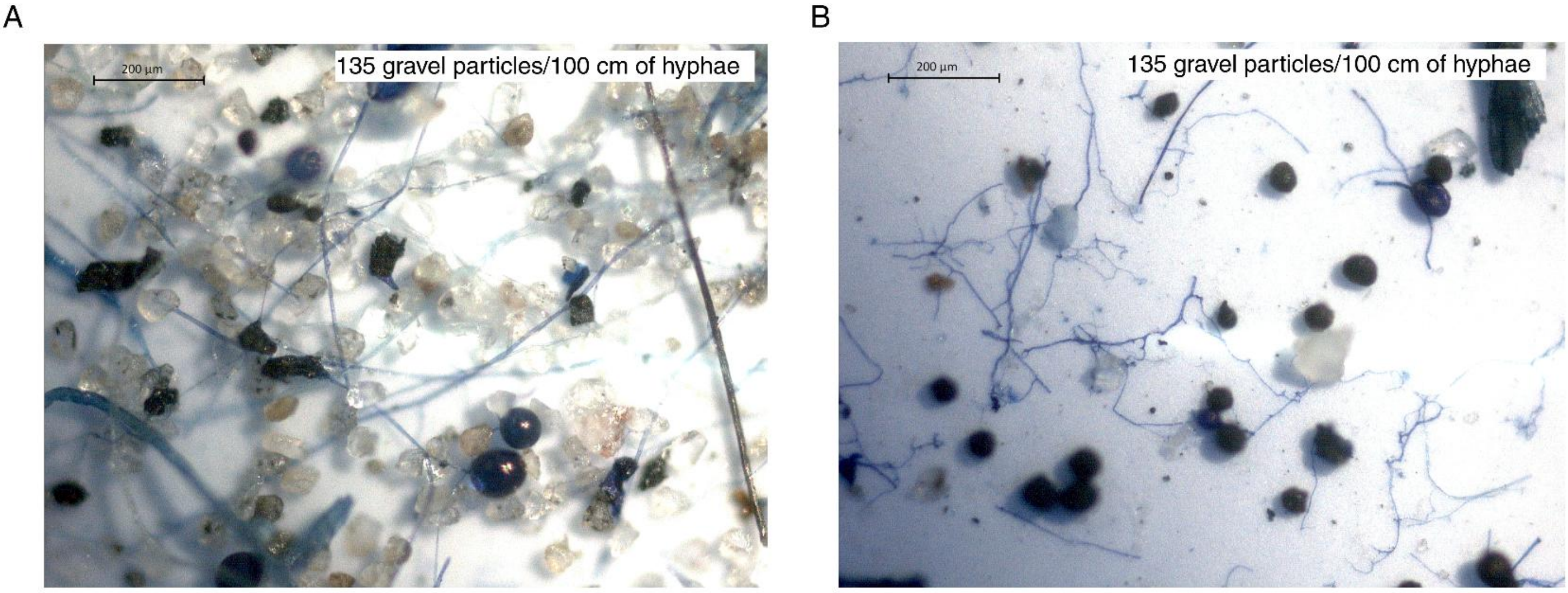

Supplementary Figure S2: Amount of residual material remaining after processing, depending on soil type. Sandy soils (A) showed higher amounts of residual material compared to soils with higher clay content (B). Numbers indicate the counted residual material (gravel) in each 2 mL hyphal extraction sample, determined by manual counting through visual inspection. Three replicates were taken, and the other two replicates showed similar results.

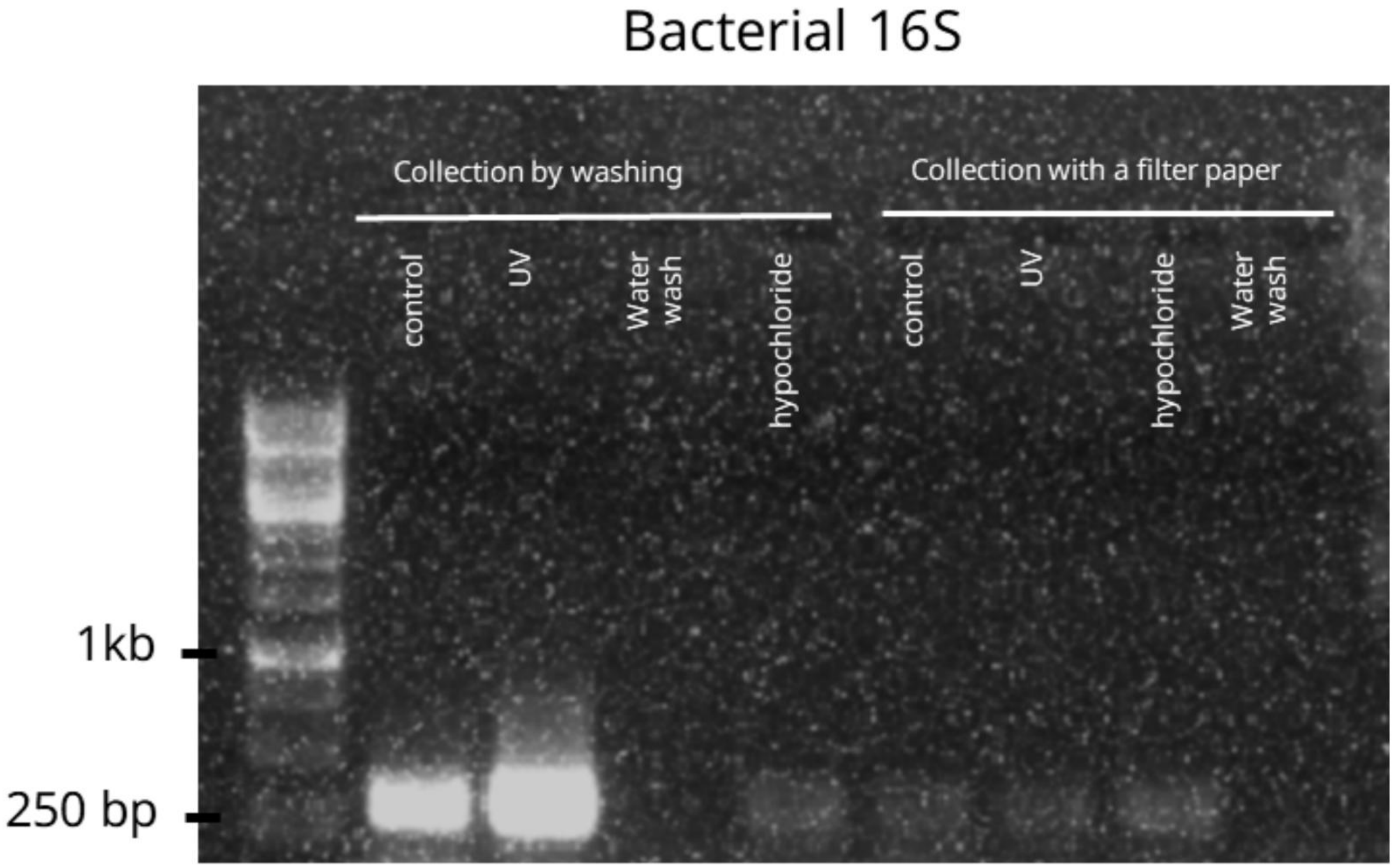

Supplementary Figure S3: Bacterial contamination on sieves was lowest when sieves were sonicated and then washed with water after a single fungal hyphae extraction. To detect bacterial contamination, PCR targeting the bacterial 16S rRNA gene was performed on sieves treated either by sonication followed by water washing, or by UV light exposure, or hypochlorite treatment. Positive controls in the leftmost lanes represent unwashed sieves.

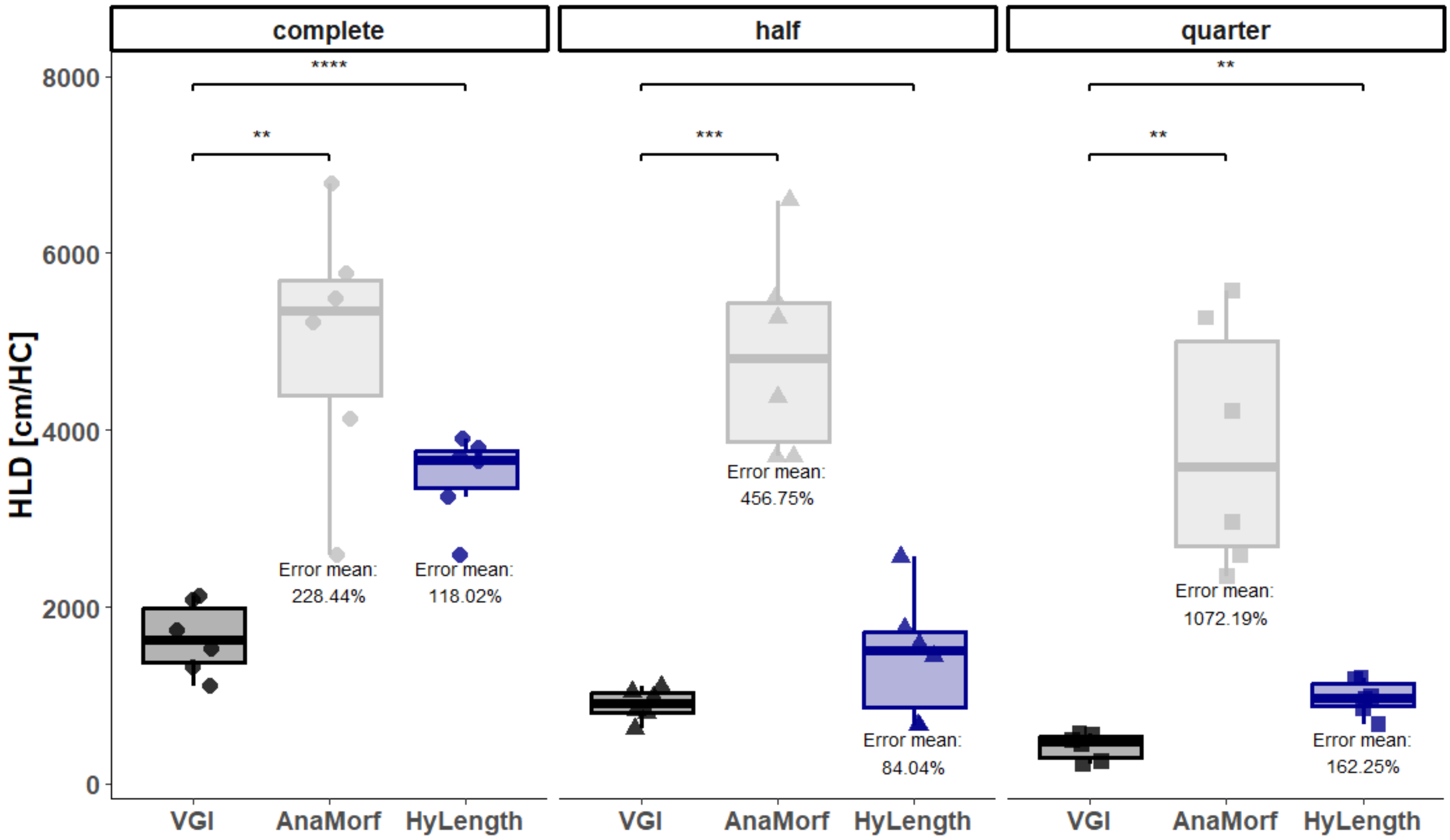

Supplementary Figure S4: Two automated image analysis methods overestimated hyphal length density (HLD). Soil containing fungal hyphae was homogenized and divided into samples of full weight (complete), half weight (half), and quarter weight (quarter). Hyphal length density per sample was then counted using the VGI method, as well as semi-automated counting using the Fiji plug-in AnaMorf (Barry *et al.*, 2009; Schindelin *et al.*, 2012) or with software HyLength (Cardini *et al.*, 2020). Statistical differences among methods were assessed by paired t-tests ($p < 0.05$). Mean percentage errors relative to manual VGI counts are displayed below each method. Significance codes: '****'0.0001 '***' 0.001 '**' 0.01 '*' 0.05 '.' 0.1 ' ' 1.

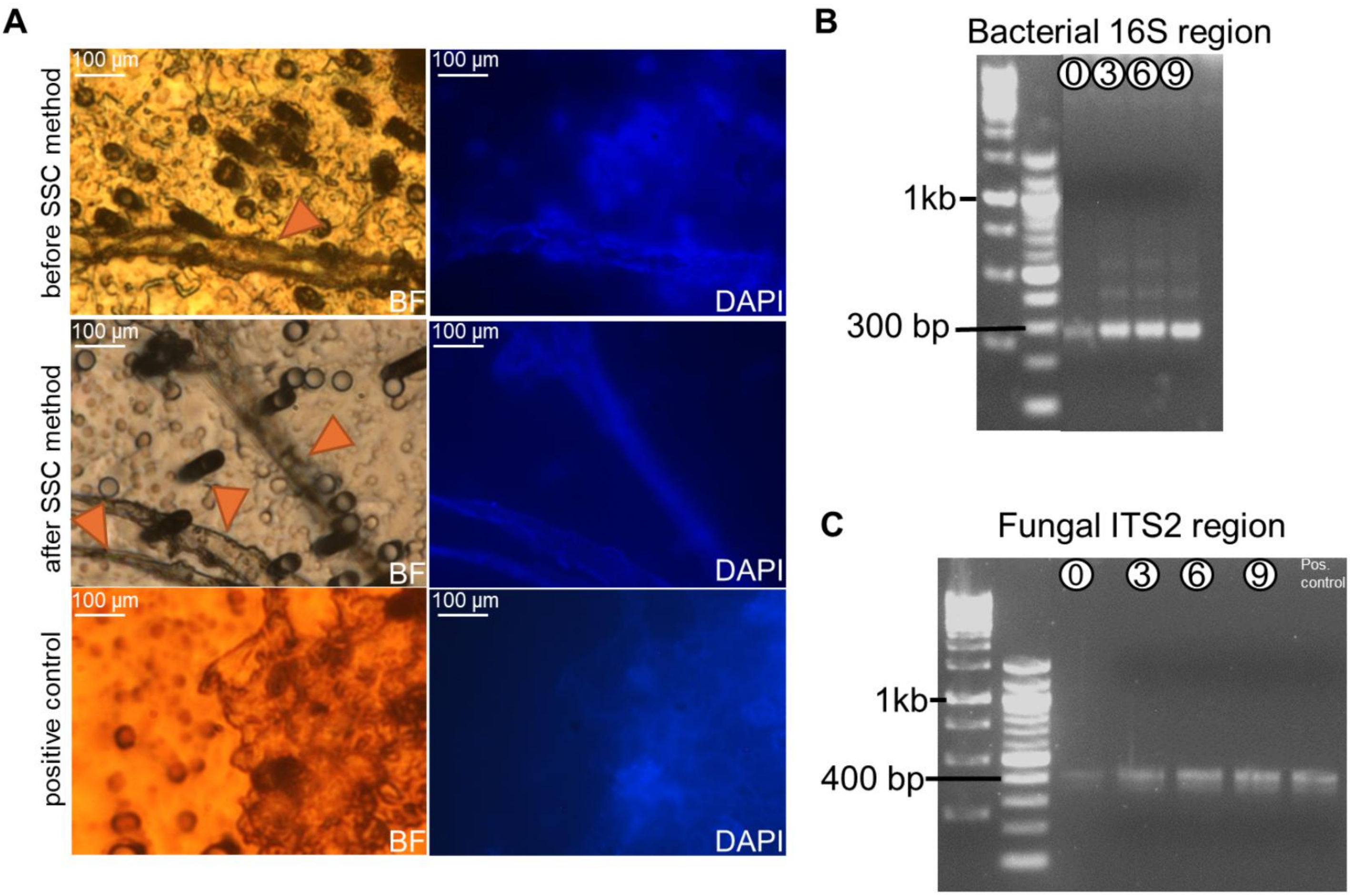

Supplementary Figure S5: Bacterial and fungal DNA can be detected using the SSC method. (A) DAPI was used to stain bacterial and fungal DNA. Staining was performed on samples containing hyphae picked with forceps from the soil of the hyphal container, samples in which hyphae and attached microbes were isolated from soil using the SSC method, and a positive control. The positive control was a cultivated hyphosphere bacterium from an agar plate. Bright-field (BF) images are shown, and DAPI was visualized with UV excitation. PCR was conducted to detect the presence of bacteria (B) by amplifying the bacterial 16S region and to detect the presence of fungi (C) by amplifying the ITS2 region of fungi. The numbers 0, 3, 6, and 9 represent different steps: before the start of the method, after the second sieving step, after the last washing step, and after freeze-drying, respectively. The lane at right shows the positive (Pos.) control.
